# Advanced Operationalization Framework for Climate-Resilient Urban Public Health Care Services: Composite Indicators-Based Scenario Assessment of Khon Kaen City, Thailand

**DOI:** 10.3390/ijerph19031283

**Published:** 2022-01-24

**Authors:** Wiriya Puntub, Stefan Greiving

**Affiliations:** Department of Spatial Planning, Institute of Spatial Planning (IRPUD), TU Dortmund University, 44221 Dortmund, Germany; stefan.greiving@tu-dortmund.de

**Keywords:** future-oriented climate impact operationalization, composite indicators, collaborative scenario planning, climate-resilient public health service

## Abstract

Conventional local public health planning and monitoring are insufficiently addressing the conjugated impact of urban development change and climate change in the future. The existing checklist and index often ignore the spatial-network interaction determining urban public health services in forward-looking aspects. This study offers and demonstrates a climate-resilient operationalization framework for urban public health services considering the interaction between urban development change and climate change across scales. A combination of collaborative scenario planning and tailor-made composite indicators were applied based on the IPCC Fifth Assessment Report (AR5)’s climate risk concept to adhere to local realities and diverse sets of scenarios. The framework was contested in a medium-sized city with a universal health care coverage setting, Khon Kaen city, Thailand. The results show that the coupling of collaborative scenario planning and composite indicators allows local public health care to operationalize their potential impact and climate-resilient targets in the future(s) in multiple service operation aspects. The scenarios assessment outcomes prove that although public health devotion can be fail-safe, achieving climate-resilient targets requires sectoral integration with urban development and health determining domains. Further exploration and disputation of the framework with a wider scale and diversified settings are recommended to enhance their robustness and universality.

## 1. Introduction

The Hyogo Framework for Action 2005–2015 prioritized safe hospitals under the World Disaster Reduction Campaign [1]. In 2015, WHO and alliance released framework and guidelines suggesting safe hospital framework and operationalization indicators based on the all-hazards approach (see more in Emergency Management in Health Care: An All-Hazards Approach [2]), as well as addressing the importance of vulnerability assessment and climate-resilient and sustainable technologies and infrastructure (e.g., Comprehensive Safe Hospital Framework [3], Hospital Safety Index [4], Operational Framework for Building Climate Resilient Health Systems [5]). In 2020, WHO disseminated a new guideline for health care facilities, WHO Guidance for Climate Resilient and Environmentally Sustainable Health Care Facilities [6]. The interventions proposed in the guidance are targeted to health care facility managers, and the checklist format does not allow comparison among facilities; however, the proposed generic interventions can be adapted for all sizes and levels of health care facilities. In addition to international guidelines, there are national toolkits for implementing climate resiliency and sustainability for health care facilities, such as the “Canadian Health Care Facility Climate Change Resiliency Toolkit” [7], Climate change resiliency indicators for health care facilities [8], and U.S. “Sustainable and Climate Resilient Health Care Facilities Toolkit” [9]. However, these climate resiliency tools are often in checklist format, instead of indices that track risk reduction in both individual health care facilities and service networks, and consider health determinants sectors beyond a health care facility’s fence. 

Composite indicators gain worldwide popularity as a tool for linking science to policy and practice, especially in climate change adaptation, climate resilience, and disaster risk management [10] (p. 198). A composite indicator is derived from compiling individual indicators into a single index based on a particular underlying model. Composite indicators are capable of summarizing the reality of complex and multidimensional phenomena, which can neither be captured by a single indicator nor directly measurable. Hence, simplicity is a key feature of composite indicators that facilitate evaluating the state of affairs, tracking progress, and ranking performance or countries over time. Moreover, composite indicators also help reduce difficulties in complex data interpretation and enable communication to policy decision makers and general audiences [10,11,12,13]. Nevertheless, the debate on the application of composite indicators never settled. The approach also receives strong methodological and pragmatic critiques, especially statistical misconception [12] (p. 3), [14] (p. 610), lack of transparency, raising false expectations, misleading policy messages, drawing simplistic policy conclusions [10] (p. 198), [11] (p. 6), missing separation of factual/analytical elements and normative judgements [15] (p. 65), and limited uncertainty analysis and reliability estimations [16]. The advantages and disadvantages of composite indicators are comprehensively discussed in Nardo et al. [11].

In disaster risk and climate vulnerability alone, Beccari [16] identified 106 methodologies used and 2298 unique variables based on 126 studies which were diverse in terms of focus (vulnerability, resilience, risk), approach (top-down, bottom-up, data collection), and scale (international, regional, national, local) [17] (p. 3).

Vulnerability is a theoretical concept that can be operationalized or assessed instead of being measured [10]. The IPCC AR5 defines vulnerability as “the propensity or predisposition to be adversely affected. Vulnerability encompasses a variety of concepts and elements including sensitivity or susceptibility to harm and lack of capacity to cope and adapt” [18] (p. 6). Vulnerability often contains tangible and intangible characteristics that challenge reducing its complexity in order to define a universal set of indicators for all levels and all hazards [19]. Despite myriad composite indicators and diversity of indicator construction methods [17] (p. 1), Hinkel [10] concluded that vulnerability indicators are only appropriate for the identification of vulnerable groups, areas, and systems, exclusively at the local scales [10] (pp. 198, 206) because vulnerability is context-specific [20] and requires a forward-looking perspective. However, it is still a challenge to operationalize a more sophisticated vulnerability assessment approach in a local context. Moreover, some intangible vulnerability characteristics are important and often involve the interplays of individual and collective perceptions and actions [21]. Thereby, it is challenging to quantify and capture them, particularly in monetary values, such as cultural or institutional aspects [19,22]. 

Although the health sector has increasingly focused on evidence-based public health for supporting decision-making processes, conventional health care monitoring and evaluation (M&E) are insufficient to address projections of health impacts under different climate and socio-economic futures [23]. A number of challenges restrain the introduction of climate resilience M&E in the health sector, such as lacking awareness and recognition of climate change impact on health outcomes, lacking long-term scenario planning and understanding of the uncertainties of climate projections and shared socio-economic pathways (SSPs), unawareness of the interaction with health determinants sectors, inadequate institution learning management, and lacking recognition of the importance of adaptation processes and outcomes [23]. Biddle et al. [24] found that most health system resilience studies only emphasize absorptive and adaptive capacities and underrated transformative capacity aspects of resilience. 

To address these challenges, we offer an innovative approach that shifts the local public health service from business as usual to a desirable climate-resilient future by combining a collaborative scenario planning framework and composite indicators instrument. This combination allows operationalization of the potential impact by including future climate trajectories and changes in health system demands driven by urban development change and the tendency of health policies. The approach also encompasses the local public health service to identify actions, layout transition steps strategically, and track achievement over time. 

This article reports the application of composite indicators to operationalize the potential impact of future urban development and climate change on public health services and its implication in a medium-sized city, Khon Kaen city, Thailand. The following Sections describe the concept of composite indicators-based potential impact assessment for local public health care (Section 2) and its application in Khon Kaen city, Thailand (Section 3). Meanwhile, Section 4 debates and manifests operationalizing a future-oriented climate-resilient scenario planning and linking spatial-network potential impact assessment in local public healthcare, as well as considering its challenges and constraints. The last Section concludes with lessons learnt and dispatches invitations for further research, which enhance the advancement and scalability of the local public health care climate-resilient operationalization. 

## 2. Materials and Methods

The composite indicator-based scenario assessment process in the study area can be laid out into four main elements, (1) scenario storylines development, (2) composite indicators development, (3) questionnaire survey and data analysis, and (4) sensitivity analysis. 

### 2.1. Scenarios Storyline Development

In current vulnerability studies, the socio-economic system is pitted against future climate change scenarios. Not only climatic factors but socio-economic changes such as demographic change, land-use change or changes in the health care system are also deeply uncertain. To determine a bandwidth of potential future conditions, socio-economic changes are to be projected through a scenario corridor in parallel with the changes in the climatic system in order to assess the future impact of climate change on future society. This is not only relevant on the global level as a basis for emission scenarios but also on the local level in order to enable decision-makers to derive tailor-made adaptation strategies [25].

In this study, the urban development change bandwidth of Khon Kaen city was portrayed according to a stakeholder future envisioning exercise and statistical analysis of possible population change and future land-use change dynamically in 2037, in response to the 20-year National Strategy (2018–2037) timeframe. In terms of climate scenario identification, the local climate-related hazards bandwidth was defined based on high-resolution (25 km × 25 km) and bias-corrected EC-EARTH projections (European Community Earth-System Model) under Representative Concentration Pathway (RCP) scenarios, provided by Hydro-Informatics Institute (HII). RCP 4.5 and RCP 8.5 were selected to exhibit intermediate and extreme climate scenarios during 2021–2050. To derive possible local climate scenarios, the climate projections were considered altogether with the local historical climate dataset of the relevant sub-watershed (provided by Thai Meteorological Department), as well as local water management rules and expert consultations. According to the local hazard profile, three hydro-meteorological hazards were examined in the analysis: fluvial flood, pluvial flood, and water supply scarcity. Hence, both urban development change scenarios and local climate change scenarios were coupled and presented to stakeholders as part of the validation process. Thereby, stakeholders preferred plausible scenarios (so-called trend scenario) and a desirable scenario were described. 

### 2.2. Composite Indicators Development

**Figure 1:**
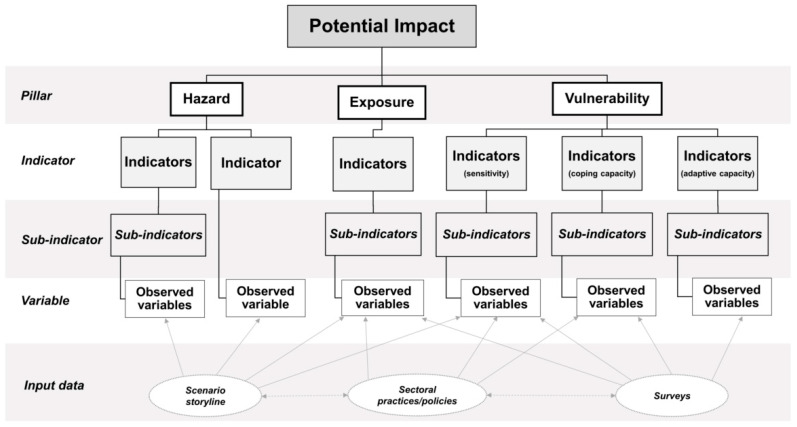
Composite indicator structure. *Source: Own illustration*.

The composite indicators are based on The IPCC’s Fifth Assessment Report (IPCC AR5) [17], where the potential impact is the non-compensable aggregation of hazard, exposure, and vulnerability (Potential impact = Hazard × Exposure × Vulnerability). The vulnerability pillar is a blending of sensitivity, coping capacity, and adaptive capacity components, which are theoretically inseparable but operationalizable [10]. Therefore, in this research, the architecture of composite indicators was structured into three hierarchical layers: pillar, indicator, and sub-indicator. The pillar layer consists of three pillars, hazard, exposure, and vulnerability. Each pillar contains subsequent indicators (indicator layer), and then each indicator is usually comprised of sub-indicator(s) (sub-indicator layer) (see Figure 1). The composite indicator development process consisted of two elements: indicator identification and normalization and aggregation.

#### 2.2.1. Indicators Identification

This composite indicators’ goal is to assess the potential impact of future urban development change and climate-related hazards change, considering their interactions with the public health service of the study area (the city). The scope of indicators was identified based on spatial and temporal dimensions of the study area, climate-related hazards management, and type of health care service and network. The indicators’ selection criteria were their relevance in describing the potential impact of the local public health service, the availability of input data, and the promotion of local public health service to operate climate-resilient targets. The future scenario storylines determine the hazard pillar and exposure pillar indicators. Meanwhile, vulnerability pillar-related indicators required a combination of the review of case studies, research papers, and relevant urban climate and disaster risk resilience [26,27,28,29,30,31,32,33,34,35], international and national public health guidelines [1,2,3,4,5,36,37,38,39,40,41,42,43,44,45,46], case studies/lessons learned [47,48,49], academic publications [50,51,52,53,54,55], as well as eight in-depth interviews with multiple public health care experts and local public health care facilities managers and executives in both national and local levels (during May 2018–March 2019). Furthermore, the health care experts survey was conducted during October–December 2019 to validate and assign weighting values for the proposed composite indicators. The experts (6) were representatives of various public health policy decision-making agencies and hospital directors/managers responsible for or those having had experiences with disaster risk management. For the hazards pillar, the experts were asked to assign the weighting value between floods and water scarcity through a budget allocation approach. Meanwhile, the ascending order prioritization technique was applied for each vulnerability-related component. Since neither the experts nor technical standards were available for justifying the weighting value of exposure-related indicators, equal weight was therefore given. It is important to note that the experts were asked to suggest additional indicators as they saw appropriate.

#### 2.2.2. Normalization and Aggregation

This study considered IPCC AR5’s climate risk concept [18] and data properties as the key criteria for selecting the normalization techniques and aggregation schemes used for each composite indicators layer (see Table 1).

- **Sub-indicator layer:** Input data were derived from both the public health care facilities’ questionnaire survey and the given scenario storylines. Distance to target normalization was applied for representing a fraction of the highest target value, which conserves the proportion and trackability of the original data. All sub-indicators under an indicator were calculated with equal-weighted arithmetic (additive) aggregation to represent an indicator.
- **Indicator level:** An indicator represents a value of at least one sub-indicator or an average value of many sub-indicators. Conceptually, individual indicators shall have a different degree of influence on the outcome of the potential impact pillar to which it belongs. Therefore, proportionately weighted normalization was used for the indicator layer; a single attribute value was divided by the sum total of the values of attributes. Weighted values can be assigned based on the experts’ judgement (hazards and vulnerability-related indicators) and equal-weighted (exposure-related indicators). In terms of aggregation scheme selection, there is a possibility that at least one of the indicator’s values may contain “0”; hence, arithmetic (additive) aggregation is operated.
- **Pillar level:** Based on the IPCC climate risk concept [18], the potential impact is a multiplication result of hazard, exposure, and vulnerability pillars. Therefore, the absence of one pillar or being assigned as 0 in one of the terms meant no potential impact occurred. To this connection, the geometric (multiplicative) aggregation method is executed for this non-compensability relationship among the pillars. In this regard, the possibility of potential impact value derived from the set of the composite indicators must be present in absolute terms between 0 to 1. A value of 0 means no potential impact, and 1 is the possible worst potential impact.

### 2.3. Questionnaire Survey and Data Analysis

Meanwhile, hazard and (partially) exposure-related indicators were obtained from scenarios storylines and the local public health facility operation practice/sectoral policies. The questionnaire acquires primary data from public health facilities fed into vulnerability and (partially) exposure-related indicators. After the test-run phase, a 35-page long questionnaire (in Thai language) consisting of 84 items was sent out to all (36) public health facilities under the Ministry of Public Health (MoPH) in the area of Mueang Khon Kaen district during December 2019–February 2020 (See Supplementary Materials, List of MoPH’s public health care facilities and questionnaire). In addition to the questionnaire survey, purposive sampling in-depth interviews with representatives of seven health care facilities from various zones of the city were conducted to capture emergent elements that might not have been reported in the survey. 

### 2.4. Sensitivity Analysis

To appraise the robustness of the composite indicators, principal component analysis (PCA) is commonly used for interpreting a relatively large series of data in a smaller number of components or dimensions that can be meaningfully interpreted while maintaining most of the variation in the dataset [56,57]. PCA can be useful for verifying the composite indicators’ robustness through a data-driven approach, especially vulnerability indicators, which comprise an extensive number of indicators compared with other potential impact pillars. A PCA was performed using Statistical Package for the Social Sciences (SPSS) software. Inspection of sampling adequacy based on the Kaiser-Meyer-Olkin (KMO) and Bartlett’s Test of Sphericity was checked. Criteria for selecting the optimal number of principal components (PCs) were either the PC had an eigenvalue greater than 1 or 70–80% of total variance was explained by all components. Reliability analysis, internal consistency within each aspect of vulnerability elements and communalities value were computed. Moreover, the structure matrix configuration was calculated to show a correlation between the variables and factors. Regarding the correlation among the PCs based on Oblimin with the Kaiser normalization rotation method, the component correlation matrix was examined to ensure a correlation among PCs. Furthermore, a standard Pearson’s correlation analysis was conducted to measure the strength of a linear correlation among multiple variables (indicators) of the vulnerability pillar. 

## 3. Results

### 3.1. Study Area—Khon Kaen City

Khon Kaen is one of the four major provinces of the northeastern region of Thailand. Mueang Khon Kaen district, the so-called Khon Kaen city, is the heart of the province and regional administration, transportation, education, medical, and economy. Mueang Khon Kaen district is the seventh most populous district in the country [58], with a size of about 953.4 square km, 416,285 (2019) registered inhabitants, 436.64 inhabitants/square km population density. Public health care in Khon Kaen city is under the umbrella of the national Universal Health Coverage Scheme. Khon Kaen Hospital’s Contracting Unit of Primary Care (CUP) network is a major public health care service network of the city consisting of 22 Sub-district Health Promotion Hospitals (SHPH), five primary care centers, and one tertiary hospital, Khon Kaen Hospital. The Khon Kaen Hospital CUP network is vital and critical for the city. As the most advanced and trustworthy hospital in the area under the full supervision of the Ministry of Public Health (MoPH), Khon Kaen Hospital has been confronting patient overcrowding as a business as a usual problem [59]. In 2020, the hospital was operated with 104% of its service capacity. Khon Kaen Hospital takes care of 75% of the workload of the CUP; the primary care service (CUP units) under the Khon Kaen Hospital CUP network share about 25% of the network workload. It is important to note that Nongbaudeemhee SHPH, Nong Ya Phraek SHPH, Donhun SHPH, and Thapra SHPH are located in Khon Kaen Mueng district area, but their operation is under a CUP network of the neighboring district.

Urbanization is not only a driver that inflicts a major challenge to ensure a balance between service capacity and service demand to Khon Kaen city’s public health care service but is also a trigger for climate-related disasters, especially floods and water scarcity. Thus, several vital questions are emerging and worth exploring with regard to how Khon Kaen city public health service could address these development challenges and climate stress simultaneously. Although Thailand has a clear and comprehensive climate change adaptation strategy at national and (some) sectoral levels, the country still lacks a framework for integrating climate resilience with long-term local development prospects and critical infrastructure operations in the city-wide perspective (see definition of critical infrastructure in Article 2, Council Directive 2008/114/EC [60]). Particularly in the public health sector, the current sectoral practice is still limited within the physical health impact landscape and a facility boundary rather than being integrated with a city-wide and service network perspective. 

### 3.2. Khon Kaen City Thailand in 2037

With stakeholder collaboration on scenarios development, the trend scenario (stakeholders preferred plausible scenario) and the desirable scenario of Khon Kaen city in 2037 under the confluence of urban development change and climate change can be articulated as follows.

#### 3.2.1. Trend Scenario Storyline

**Figure 2:**
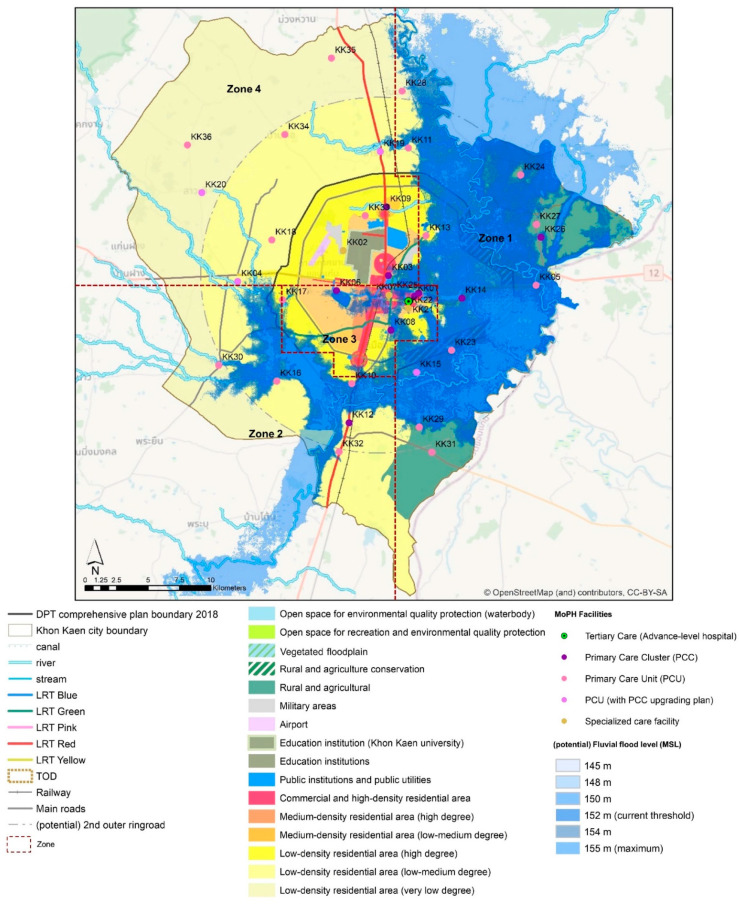
Visualization of Trend scenario. Source: Own illustration.

**Figure 3:**
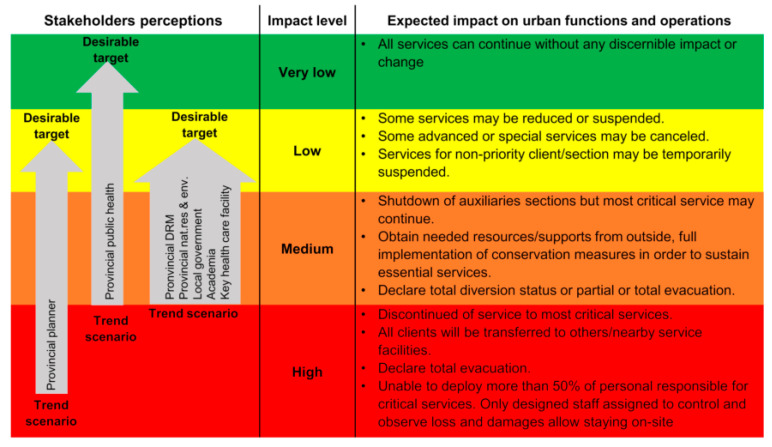
Potential impact acceptability (under trend scenario) and desirable target(s) in terms of system functions and operations. Remark: The impact level classification was inspired by [41]. Source: Own illustration.

With a clear signal of urbanization, Khon Kaen city expected about 3.6 % (on average) of population growth annually. However, the boundary between urban and rural settings is ambiguous. Expansion of low residential areas can be observed in all directions, except the eastern part of the city, where urbanization might be limited at the right edge of the first outer ring road. In responding to smart city strategies, compact commercial center and denser residential areas can be expected in the city center and TOD (transit-oriented development) nodes along with the light rail transit (LRT) structure. In this future scenario, fluvial flood at 155 m mean sea level (MSL) can be expected to affect the eastern part of the city and may interrupt activities in the public administration quarter. Khon Kaen Hospital and several primary health care units could potentially be flooded or isolated due to water blockage accessibility. Nonetheless, even though water supply scarcity is invisible, a clear trend can be anticipated as a major underlying threat to city development and its public health service. Figure 2 visualizes the coupling of the future land use change and climate-related hazards change under the “trend scenario”. In the trend scenario, Khon Kaen city’s stakeholders wish to be able to protect the commercial and business district (CBD) (see Supplementary Materials; spatial extension of the protective target) at all costs. Meanwhile, from an urban system operation point of view, the city should minimize the potential impact from a low level to a very low level (see Figure 3). 

#### 3.2.2. Desirable Scenario Storyline

The “desirable scenario” was developed based on the main features of urban development and climate-related hazards described under the “trend scenario”, which integrated both stakeholders preferred city-wide and public health-specific spatial planning-based measures. The purpose of the “desirable scenario” aims to manifest efforts of the city in minimizing the potential impact in response to the target(s) bandwidth, low to very low level (Figure 3), through multiple measures implemented directly by the public health sector and indirectly through outcomes of city-wide spatial planning-based measures as follows. 

**Figure 4:**
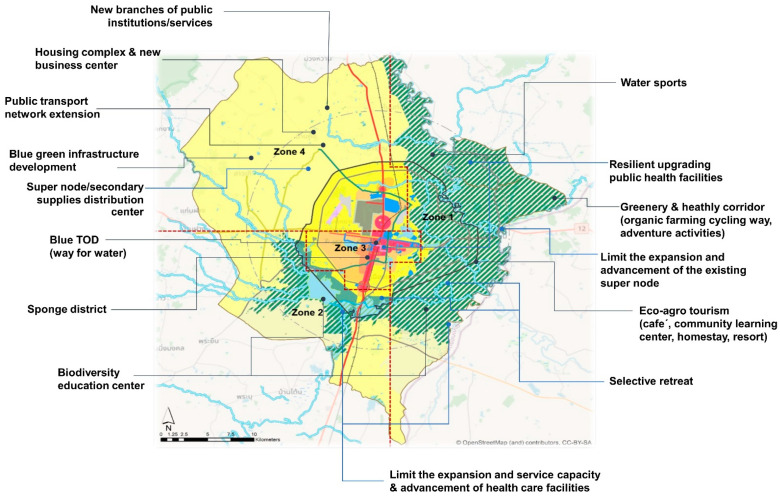
Visualization of stakeholders preferred spatial planning-based measures for minimizing the potential impact of climate-related hazards under the desirable scenario. Source: Own Illustration.

To limit urbanization in the flood-prone areas (Zones 1 and 2, eastern and southern parts), promoting agro-eco tourism and environment-friendly recreation activities will be core market-driven strategies to boost the local economy while protecting agricultural areas’ function as part of the city’s flood retention approach. Systematically addressing the possibility of 155 m MSL fluvial flood level, a hazard map is widely utilized as a key instrument for risk communication and participatory decision-making. As an outcome of these city-wide actions, the slow pace of development and population growth could contribute to relatively low health care service demands; there is no need for further extension of service capacity and the advancement of services of the existing public health units or establishing new public health care infrastructure in flood-prone areas. Nevertheless, public health units exposed to fluvial floods should be mandatory and should implement resilient upgrading and retreat strategies. Conversely, promoting blue–green infrastructure development could help mitigate hazards, reduce the number of direct injuries and loss of life due to flood risk, and enhance the local population’s environmental health and well-being, reducing service demands and costs of the health care sector and society. Densification and mixed-use magnetize more economic activities and people to the inner-city areas (Zone 3). Thereby, demand for public health services is anticipated to rise in response to this socio-demographic change in this strong urban setting. Although increasing permeable surface, improving a drainage system, and addressing water retention could cut the peak of inundation, it is fair to state that the success of the city-wide measures cannot be guaranteed. Thus, under fluvial flood events, Khon Kaen Hospital and other healthcare facilities in this zone can be isolated or surrounded and hampered by floods. As a safer area (Zone 4), the city will invest in public infrastructure and livability in order to serve the increasing demands of new inhabitants and economic activities. However, the local public health service, instead of establishing new public health facilities or increasing the advancement of the existing care units to respond to the increasing service demands, assigning an existing high capacity service unit to be a supernode or secondary medical supply center could be a practical alternative. Figure 4 visualizes stakeholders’ preferred spatial planning-based measures for the desirable scenario.

### 3.3. Composite Indicators Development

According to IPCC AR5 [17], the composite climate risk indicators are structured into three pillars that consist of 3 hazard indicators, 2 exposure indicators, and 17 vulnerability indicators, see Supplementary Materials, description and justification of indicators.

**Hazard indicators** were determined based on climate-related hazards bandwidth. Thus, flood (H1) and water scarcity (H2) were assigned as hazard-related indicators. This research further categorized sub-indicators as fluvial flood (H1.1), pluvial flood (H1.2), and water scarcity (H2.0) according to the local climate-related hazard profile. The input data were derived from geospatial analysis of climate-related hazards scenario bandwidths. 

**Exposure indicators** were characterized based on the presence or location of public health care facility’s buildings (E1) and the position of their vital working systems (E2). Exposure of buildings is distinct among the three considered climate-related hazards based on the possible future climate-related hazards bandwidth, fluvial flood (E1.1), pluvial flood (E1.2), and water supply scarcity (E1.3). Exposure of working system (E2) can be further broken down to the location/position of primary working systems (E2.1) and location/position of reserved (secondary) working systems (E2.2). The input data were derived from geospatial analysis of climate-related hazards scenarios bandwidths as well as a questionnaire survey of the local public health facilities.

**Vulnerability indicators** were articulated into three core elements: sensitivity (or susceptibility), coping capacity, and adaptive capacity. 

#### 3.3.1. Sensitivity

This study implies sensitivity as a state of public health care service and operation is easily affected or reacts to urban development change and climate-related hazards stimuli given to the properties, function, and goal of the system. Over carrying capacity, variety of vulnerable patients, deficits of available resources, and malfunction/disruption of the working system could make public health services likely to be fragile and adversely affected by urban development change and climate-related hazards. Moreover, the public health sector also highly depends on and is interconnected with other critical infrastructures and urban domains. Altogether with literature [1,2,3,4,5,36,37,38,39,40,41,42,43,44,45,46,47,48,49,50,51,52,53,54,55] and discussions with the local health care experts, sensitivity elements of vulnerability indicators were characterized into five major indicators: over carrying capacity (V1); variety of vulnerable patients (V2); resource insufficiency (V3); poor system conditions and maintenance of working systems (V4); and downtime of essential working systems (V5). This set of indicators comprises nine sub-indicators structured based on the input data derived from the survey combined with public health sector policy and practices. 

#### 3.3.2. Coping Capacity and Adaptive Capacity

This study streamlined the capacity component of the vulnerability pillar as lacking key attributes that enhance climate resilience. The capacity-related indicators consist of 12 indicators and 40 sub-indicators structured based on input data derived from the survey combined with public health sector policy and management schemes. These composite indicators can be grouped as six coping capacity-related elements (i.e., flexibility and modularity (V6); diversity of suppliers (V7); redundancy (V8); responsiveness (V9); resource mobilization (V10); and integration and coordination (V11)) and six adaptive capacity-related elements (i.e., information (V12); preparedness and risk transfer (V13); participation and inclusiveness (V14); capacity development (V15); mainstreaming climate-risk in planning process (V16); and monitoring and evaluation (V17)). The identified composite indicators represent both spatial and network dimensions of the local public health service. It also reflects key properties and characteristics of an overall working system and dependency and interdependency among units of the Khon Kaen Hospital CUP network as well as the whole public health service network with other health determinant sectors. 

### 3.4. Dataset and Normalization

A total of 25 completed questionnaires (or about 69%) were received from one tertiary care hospital, 21 primary care units (PCUs), and three specialized facilities. Among these, one tertiary care is Khon Kaen Hospital CUP host, and 19 PCUs are members of the Khon Kaen Hospital CUP network. It is important to note that the three specialized facilities are independently operated under MoPH. Five interviews with the local public health care facilities managers/executives were conducted to contextualize local realities of various zones of the city. Distance to target normalization was applied for unifying scales and units of the input dataset. Every sub-indicator was calculated with equal-weighted arithmetic (additive) aggregation in order to represent their respective indicator. At the indicator level, an indicator represented a value of at least one sub-indicator or an average value of many sub-indicators. The indicators values were then arithmetically aggregated with proportionately weighted normalization to represent pillar values. According to the mathematical operation of the climate risk concept, the geometric aggregation with proportionate normalization was calculated for this non-compensability interaction among potential impact pillars. Hence, this study classified the potential impact outcomes (0 to 1) in four equal intervals that reflect each composite indicator pillar and ensure trackability of the outcomes: very low (0.000–0.016), low (>0.016–0.125), medium (>0.125–0.422), and high (>0.422–1.000). 

### 3.5. Potential Impact Analysis

Under “trend scenario”, the potential impact of the local public health services is at medium level, from the area-based perspective. In the network-based aspect, although the Khon Kaen Hospital (CUP-host) and PCUs under the network (CUP-units) (on average) revealed almost the same hazard pillar value, the CUP host obviously showed lower exposure and vulnerability than its CUP units (on average). As a result, a network operation of public health service could theoretically influence the potential impact of climate-related hazards of Khon Kaen city. Note that this analytical result was based only on the survey of participating public health facilities; therefore, about 93% of the CUP network was illustrated through this research result. 

The result of the “desirable scenario” showed that direct implementation of the public health care sector could reduce the potential impact level from medium level (under the “trend scenario”) to low level (under the “desirable scenario”). However, suppose the local public health service would like to achieve a “very low” level of potential impact minimization target. In that case, more efforts shall be put not only in their internal exposure and sensitivity-related vulnerability elements but also require city-wide actions on hazard mitigation and exposure avoidance. Nonetheless, neither successful nor effective outcomes of the city-wide measures can be guaranteed. Thus, this research highlights a conservative assumption that the local public health service would deal with the potential impacts through self-reliance orientation. Table 2 illustrates the potential impact assessment of the trend scenario and desirable scenario in both area-based and network-based perspectives.

### 3.6. Sensitivity Analysis

Based on the composite indicator structure, the PCA technique was applied for the vulnerability pillar, which consists of 17 indicators or variables. According to the questionnaire responses, the sample size for this statistical analysis was 25 (out of 36). The vulnerability pillar’s overall mean value is 0.42, and the standard deviation is 0.16. Average Cronbach’ alpha values = 0.852, which is considered to be good internal consistency (>0.7). Inspection of sampling adequacy based on the Kaiser-Meyer-Olkin (KMO) value of 0.524 is miserable but still acceptable for PCA execution. Bartlett’s Test of Sphericity, chi-square approximation is 268.26, *p* < 0.001. The result shows that five PCs represented 75.781% of the total variation, as well as obtaining eigenvalues greater than 1. A scree plot also confirmed the choice of five PCs, with total variance explained (eigenvalues of which are great than 1). Considering reducing variables, the indicators that have factor loading less than |0.4| on either PC1 or PC2 should be dropped out. In this regard, the PCA suggested excluding V2, V4, V5, V6, V7, and V10 from the analysis; the result still holds a meaningful interpretation. Nevertheless, these indicators still show strong factor loading in other PCs (PC3, PC4, and PC5), which shared about 25% explained variance in the result. According to Pearson correlation analysis, the result shows that all the indicators (independent variables) are positively correlated with the vulnerability pillar score (dependent variable).

Even though there is no universal rule of thumb for corrected item-total correlation value, values that are below 0.3 [61] or 0.2 are undesirable. Despite small sample size, this data-driven analysis strongly suggested that the set of sensitivity-related indicators shall be improved to ensure better distribution of the dataset. Coping capacity and adaptive capacity-related indicators showed a good Cronbach’s alpha coefficient value (0.80 and 0.85, respectively). Moreover, all coping capacity-related indicators presented a good positive inter-item correlation value and no suggestion to delete items for better internal consistency except V7 (lack of diversity of suppliers), which exhibited low and negative inter-item correlation value. Therefore, PCA suggested improving the reliability and internal consistency of the coping capacity aspect of the vulnerability pillar by removing V7. Meanwhile, adaptive capacity-related indicators illustrated good inter-item correlation values; every item obtains an inter-item correlation greater than +0.3. Nonetheless, V12 (lack of information) showed a relatively small, corrected item-total correlation, but the difference between the item’s Cronbach’s alpha and the overall Cronbach’s alpha is negligible (+0.003). Therefore, it is fair to conclude that adaptive capacity-related indicators are internally consistent. Detials information regarding PCA analysis can be found in Supplementary Materials, sensitivity analysis i.e., KMO and Bartlett’s test, Scree plot, Total variance explained, Communalities value, Pattern matrix, Scatterplot of rotated loading factors (pattern matrix), Structure matrix, Component correlation matrix. 

## 4. Discussion

This paper demonstrates operationalizing future-oriented climate potential impact assessment in Khon Kaen city that underlined interlinkages of spatial and network dimensions in local public health service and intertwined its urban development. The following thoroughly discuss gaps and challenges in utilizing composite indicators as a tool for operationalizing the climate-resilience future of local public health based on the IPCC AR5 climate risk concept. 

### 4.1. Composite Indicators-Based Scenario Assessment—A Novel Tool for Climate-Resilient Health Care Services 

The future potential impact of the local public health care units mainly depends on city-wide hazard mitigation in both structure and non-structural measures. The case of Khon Kaen city showed that even though no actions are implemented at the city-wide level, maximizing local public health care endeavors to reduce vulnerabilities and internal exposures help maintain its function with certain residue impacts or fail-safes. Nonetheless, to minimize the potential impact to a negligible level as an upper-tier target, immense sectoral change in unleashing structural deadlocks in terms of personnel and resources management is a prerequisite, on the one hand. On the other hand, the planning paradigm needs to be shifted from considering a relatively short-term demographic change and emergency response to longer scenarios planning considering future-oriented changes in demo-socioeconomic, urban development, and climate-related hazards. In addition to planning orientation, when coming to climate-related hazards, the mindset of public health care service in Thailand needs to be reshaped from going out and helping other people first to the hospital having to be safe first because the hospital as critical infrastructure would be able to continue its operation and serve as a community lighthouse in the crisis. These shifts and changes have to mainstream throughout public health domains and local public health care networks. Moreover, it is pivotal to ensure cooperation with health determining sectors, especially other critical infrastructures and local governments, in managing and investing in climate resilience. Consideration of the criticality of public health service-dependent lifeline infrastructures is also very important to understand and operationalize, with possible cascading effects on local public health services and beyond. This is because a serious deterioration or complete disruption of health care could affect the operability of infrastructures that are not directly exposed to climate risks. Finally, the sickness rate is an important factor for business continuity; therefore, a lack of health care service would automatically increase sick leaves.

In a country where public health care has to operate at the margin of resources, investment in potential impact mitigation with a long-term perspective is a great challenge and hindered by short-term problems and emergency constraints. However, visualization and articulation of scenarios are insufficient. Detailed cost-benefit analysis or risk-aversion estimation and feasibility studies on implementation options are highly required. Reaching the desirable climate-resilient targets demands tremendous efforts that may exacerbate underlying problems of Thai’s public health system, especially understaffing and capacity overload of tertiary care. Nonetheless, emphasis that the central procurement system oversight by the Ministry of Finance hinders public health service in being prepared and ready for the future development and climate change challenges. Therefore, climate change coordination units under the public health ministry and local universities could play a vital role in setting up the reporting system and capacity-building activities, facilitating and keeping potential impact assessment in check. Thus, responsible agencies should consolidate the composite indicators with the existing safety standard and monitoring and reporting system in order to avoid overwhelming a local health care unit with the additional burden from their routine works. Importantly, the application of the composite indicators in Khon Kaen city exhibits a great opportunity for upscaling and conforming to the framework as a standard self-assessment and benchmarking scheme, which is useful for both facility-based and network-based public health care operations. 

### 4.2. Constraints and Scalability Opportunities

The application of the composite indicators emphasizes the necessities of specific technical debate on limitations and scalability potential of the indicators framework as follows. 

#### 4.2.1. Sectoral Benchmarking in Needs

Even though the relevant experts had reviewed the structure of composite indicators, further and deeper discussions are required to improve the indicators’ reliability and internal consistency, especially exposure, sensitivity, and coping capacity-related indicators. Some input data require a debate on a certain benchmark among public health domains, such as critical downtime of essential working systems, level of redundancy, a ratio of vulnerable patients, etc. In this regard, it is essential to organize workshops with experts in order to justify or reach consensus on appropriate definitions, characteristics, and weighting schemes of indicators and sub-indicators, hence deriving a sectoral or national benchmarking, which could upscale to be a national/universal guideline. Moreover, a wider contest of composite indicators with larger sample size is vital for selecting appropriate weighting techniques, e.g., expert-weighted, equal-weighted, data-driven. These improvements should benefit for upscaling and developing this set of composite indicators as a national or international standard.

#### 4.2.2. Data Constraints and Statistical Conciseness

This study demonstrates fundamental challenges in performing a future-oriented composite indicators-based potential impact assessment on the basis of the IPCC AR5 climate risk concept. Three aspects that reflect the robustness of the composite indicators and the reliability of the potential impact outcomes are balancing composite indicator structure, statistical and theoretical convergence, and quality of input data.

- **Balancing the number of indicators**

The indices applied in this potential impact assessment were fitted for the purpose. The number of indicators and sub-indicators depends on the local climate-related hazard profile, urban development context, and public health service characteristics. Imbalance is clear, particularly the vulnerability pillar, with 49 sub-indicators derived from 223 input data hidden behind a single number. Although this study applied distance to target and proportionate normalization schemes to conserve the complexity of the crucial properties of the system as well as promote M&E of the potential impact mitigation target(s), communication of the result should be very mindful of the possible obscuring of visibility of public health efforts which, mostly laying under the vulnerability pillar.

- **Statistical and Theoretical Convergence**

The nature of input data may play a major role in this statistical and theoretical dissonance. The majority of input data representing adaptive capacity-related indicators are homogenous in the form of checking of the availability and implementation status of relevant plans and policies, which enable transformation change or enhance capacity in a long-term perspective. Since climate/disaster risk management is not mainstream to local public health policy and operation in Thailand, most participating hospitals fed almost the same figure of input data or presented almost no variation in the dataset. In contrast, sensitivity and coping capacity-related indicators illustrate distinctly heterogeneous datasets based on the day-to-day operation of the local public health units. These contradictions reflect low reliability and internal consistency of sensitivity and coping capacity-related indicators compared with adaptive capacity-related indicators. In addition, finding statistical convergence with the theoretical influence of scenario-based composite indicators is challenging, especially in dealing with input data polarization in responding to the scenarios’ storylines. For example, the “trend scenario” condition assumed that all hospitals might deal with an imbalance between service demand and supply. Therefore, all of them exhibited the identical figure for this sensitivity-related indicator; in other words, no variance in the dataset. 

Furthermore, even though almost 70% of the questionnaires received is considered to be a high return rate, sample sizes of only 25 are seriously challenging for applying data-driven sensitivity analysis techniques. Although the analysis result shows an acceptable ratio of sampling adequacy, a larger sample size is preferable [62]. Therefore, expanding the study areas to provincial or national scale is recommended to improve the robustness of vulnerability indicators. Additionally, these statistical concerns may prevent utilizing data-driven approaches as weighted normalization schemes to ensure the robustness of the composite indicators’ outcomes.

- **Quality of Input Data**

In addition to the complexity of the survey, the respondents’ understanding of the questions and their knowledge and awareness of socio-economic development and disaster risk management is crucial to ensure the input data’s consistency, especially on the issues beyond their day-to-day routine or area of responsibility. Moreover, the eligibility of the survey respondents is also vital. Although, in this study the survey addressed the director/head of a service unit, a few primary care units assigned a professional nurse to answer the questionnaire. Undoubtedly those nurses are the right person to provide input data regarding day-to-day operation issues; however, questions regarding policy and long-term planning are often beyond their ability to answer. Therefore, this study suggests organizing an orientation workshop with the responders to ensure the quality of input data and the implications of the assessment. Additionally, answering the questionnaire is more challenging at a higher health care service level, especially in tertiary care or specialized care; thereby, coordination among relevant departments is essential. Furthermore, this study found that interviews with local public health facilities are helpful to obtain valuable input that the questionnaires may not be able to capture, e.g., the dilemma of human resource management and procurement regulations. 

## 5. Conclusions

This research suggests a novel framework for operationalizing local public health care under the two future global mega challenges: urbanization and climate change. The composite indicators-based scenario assessment was developed on the foundation of the IPCC AR5 climate risk concept. It assembles scenario planning and composite-indicator tools to fasten the day-to-day public health operation and long-term strategic planning aspect, which public health care operators and policymakers always ignore. The composite indicators provide a broader and deeper perspective of potential impact and promote spatial-network integration with other health determining realms that are more advanced than the existing practices. Moreover, configurations of the potential impact outcomes can be manifested to fit the needs of different policy decision-making levels, such as from the individual health care facility, area-based, service network, and service level-based points of view. 

Experience in Khon Kaen city shows that addressing the interaction between urbanization and climate change and its impact on urban public health services is indispensable, and that the existing checklist is insufficient. Thereby, this research confirms that the offered composite indicators approach is appropriate in operationalizing the complexity of the potential impact of climate-related hazards on public health services. Furthermore, it is also simple enough for a public health facility’s manager or staff to conduct this self-assessment without extensive technical support. Nevertheless, there are two emerging concerns on the assessment outcomes and the dilemma of statistical and theoretical convergence. 

Through the composite indicator-based operationalization of the IPCC AR5 climate risk concept algorithm, communication of the potential impact outcomes should be very mindful of the possible bind faced by public health actions when reducing vulnerability and avoiding internal exposure. Despite the possibility that public health care actors might invest immense effort in changing and transforming their working systems or operations, the outcomes in mitigating potential impact may be insignificant without citywide actions on hazards mitigation and exposure minimization. However, this highlights opportunities to promote integrative planning between the local public health care sector and other urban domains in executing climate resilience interventions. 

In addition, balancing a number of composite indicators at the pillar level to reflect reality and the alteration of input data so that they adhere to scenario storylines raises statistical robustness challenges for the composite indicators in this study. With a limited sample size within the scope of the study area, the robustness of the composite indicators should be further explored with a larger spatial scope and diversity of local settings and hazard profiles to strengthen statistical and theoretical convergence and to ensure the universality of the findings. Nonetheless, this study emphasizes the necessity of benchmarking specific indicators to reinforce the scalability of the composite indicators as the national standard, while considering regional and local differences. 

Vitally, the composite indicators shed light on the interface of the local public health care with climate-related hazards and the urban development dynamic, rather than engineering aspects of working systems or taking technology forecasts into account. Thereby, to develop a more advanced version of the composite indicators, it is valuable to consider the criticality and socio-technological aspects of working systems of a health care service network and its relevant critical infrastructure. 

## Figures and Tables

**Table 1:** Normalization, weighting, and aggregation used for composite indicator-based potential impact assessment in this study.

Composite Indicator Layers	Aggregation Schemes	Weighting Schemes	Normalization Schemes
**Pillar**	Multiplicative	Equal weight	Proportionate normalization
**Indicator**	Additive	Equal weight and Expert weight	Proportionate normalization
**Sub-indicator**	Additive	Equal weight	Distance to target normalization

**Table 2:** Potential impact assessment of Khon Kaen city in area-based and service network perspectives.

Indicators	Trend Scenario		Desired Scenario	
	Area-Based	Service Network	Area-Based	Service Network
H1: Fluvial flood	0.588	0.639	0.588	0.639
H2: Pluvial flood	1.279	1.279	1.279	1.279
H3: Water scarcity	1.442	1.442	1.442	1.442
**Weighted normalized Hazard**	**0.827**	**0.840**	**0.827**	**0.840**
E1: Exposure of public health facility’s building(s)	1.040	1.017	1.040	1.017
E2: Exposure of working systems	1.594	1.178	0.263	0.279
**Weighted normalized Exposure**	**0.659**	**0.549**	**0.326**	**0.324**
V1: Over carrying capacity	0.800	0.800	0.480	0.710
V2: Variety of vulnerable patients	0.683	0.778	0.683	0.778
V3: Resource insufficiency	0.624	0.608	0.440	0.555
V4: Poor system conditions and maintenance of essential working systems	0.064	0.033	0.000	0.000
V5: Downtime of essential working systems	0.053	0.045	0.053	0.045
V6: Flexibility and modularity	0.456	0.480	0.000	0.000
V7: Diversity of suppliers	0.217	0.122	0.047	0.031
V8: Redundancy	0.416	0.397	0.081	0.097
V9: Responsiveness	0.626	0.559	0.000	0.000
V10: Resource mobilization	0.359	0.535	0.000	0.000
V11: Integration and coordination	0.300	0.084	0.000	0.000
V12: Information	0.388	0.424	0.000	0.000
V13: Preparedness and risk transfer	0.720	0.594	0.000	0.000
V14: Participation and inclusiveness	0.742	0.808	0.000	0.000
V15: Capacity development	0.642	0.167	0.000	0.000
V16: Mainstreaming climate-risk in planning process	0.440	0.487	0.000	0.000
V17: Monitoring and evaluation	0.300	0.323	0.000	0.000
**Weighted normalized Vulnerability**	**0.461**	**0.426**	**0.105**	**0.130**
**Potential impact = HxExV** Very low (0.000–0.016) Low (>0.016–0.125) Medium (>0.125–0.422) High (>0.422–1.000)	**0.251**	**0.196**	**0.028**	**0.035**

## Data Availability

Data presented in this study are available in the Supplementary Materials.

## Supplementary Materials

The following are available online at https://www.mdpi.com/article/10.3390/ijerph19031283/s1, Figure S1: Spatial extension of the protective target under the trend scenario defined by the stakeholders, Figure S2: Scree plot, Figure S3: Scatterplot of rotated loading factors (pattern matrix), Table S1: List of MoPH’s public health care facilities, Table S2: Details and justifications of hazard pillar indicators, Table S3: Details and justifications of exposure pillar indicators, Table S4: Details and justifications of vulnerability pillar indicators, Table S5: KMO and Bartlett’s test, Table S6: Total variance explained, Table S7: Communalities value, Table S8: Pattern matrix, Table S9: Structure matrix, Table S10: Component correlation matrix, File S1: Questionnaire [63,64,65,66,67,68,69,70,71,72,73,74,75,76,77,78,79,80,81,82,83,84,85,86,87,88,89].
