# Supplementary material for: Advanced Operationalization Framework for Climate-Resilient Urban Public Health Care Services: Composite Indicators-Based Scenario Assessment of Khon Kaen City, Thailand
Source: Int J Environ Res Public Health. 2022 Jan 24;19(3):1283. doi: 10.3390/ijerph19031283 (PMC8834807; doi:10.3390/ijerph19031283)
Supplement: Supplementary file 1 [file ijerph-19-01283-s001.zip › ijerph-1523465-supplementary.pdf]

International Journal of
Environmental Research and Public Health

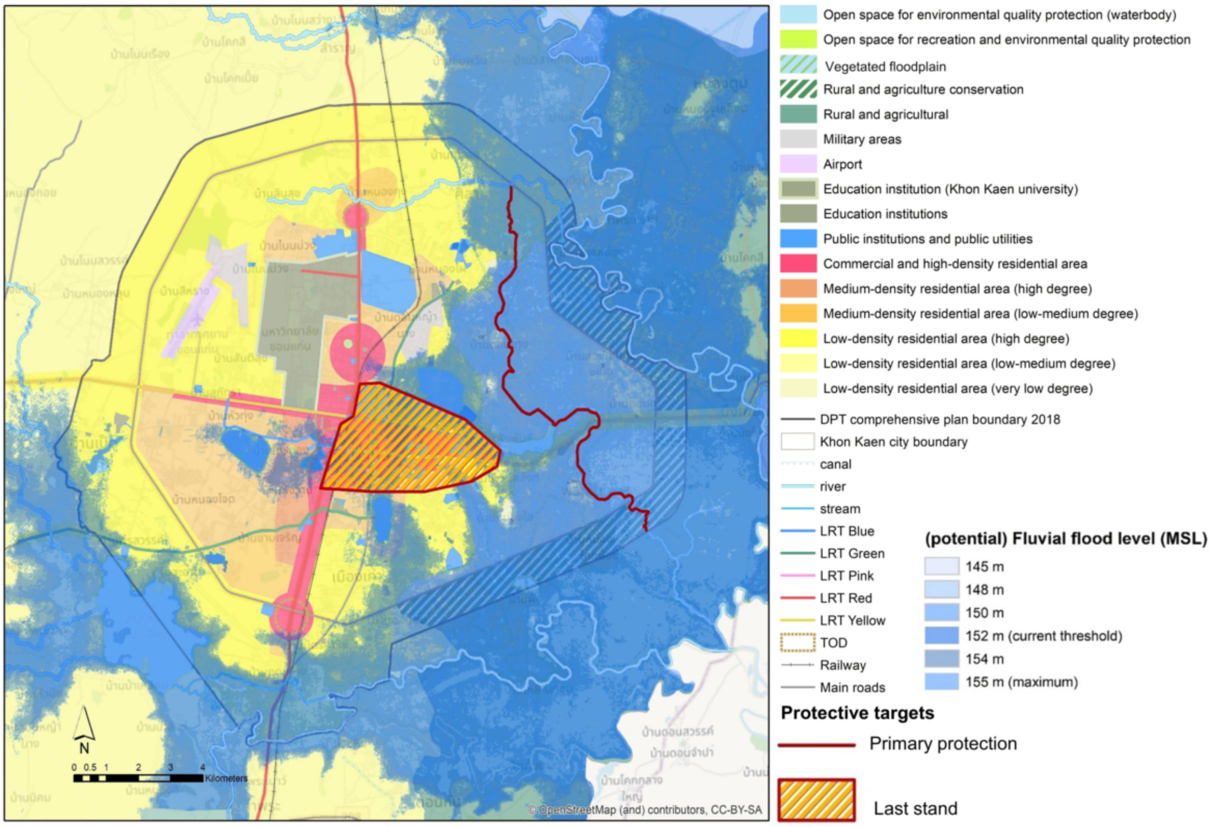

Figure S1. Spatial extension of the protective target under the trend scenario defined by the stakeholders.

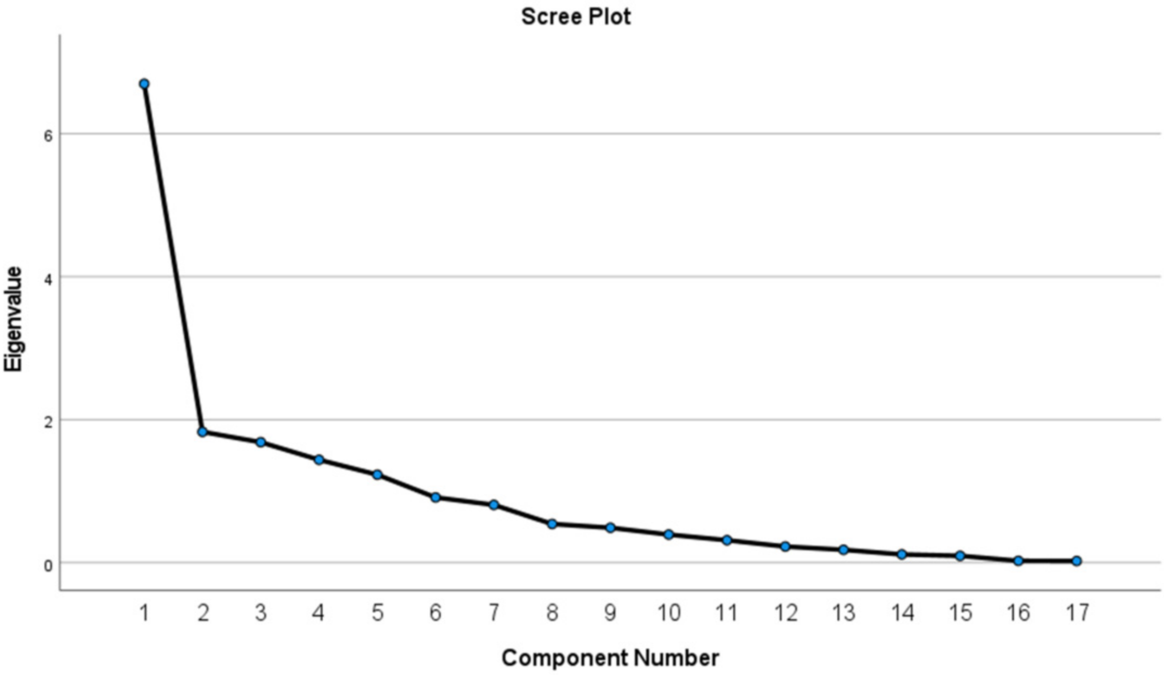

Figure S2. Scree plot.

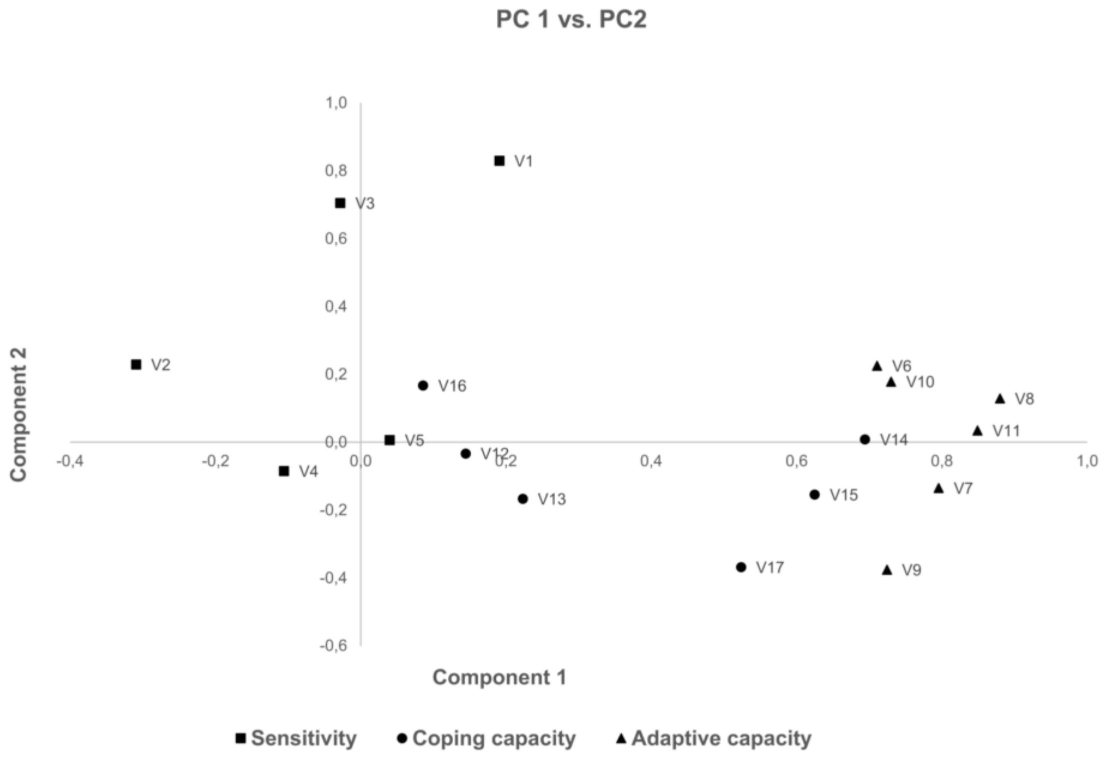

Figure S3. Scatterplot of rotated loading factors (pattern matrix).

Table S1. List of MoPH's public health care facilities.

Location	Code	Public Healthcare Facility	Service Hierarchy	KKH CUP network	Questionnaire respondent
Zone 1	KK05	Beungniam Sub-district Health Promotion Hospital	Primary Care Unit	Yes	Yes
Zone 1	KK11	Ban Kok Sub-district Health Promotion Hospital	Primary Care Unit	Yes	Yes
Zone 1	KK13	Sila Sub-district Health Promotion Hospital	Primary Care Unit	Yes	Yes
Zone 1	KK14	Pralub Sub-district Health Promotion Hospital	Primary Care Unit	Yes	Yes
Zone 1	KK23	Ban Phue Sub-district Health Promotion Hospital	Primary Care Unit	Yes	Yes
Zone 1	KK24	Nong Toom Sub-district Health Promotion Hospital	Primary Care Unit	Yes	Yes
Zone 1	KK26	Khon Kaen Hospital 2 (Sirithammikawat Temple Branch)	Primary Care Unit	Yes	**No**
Zone 1	KK27	Koksi Sub-district Health Promotion Hospital	Primary Care Unit	Yes	No
Zone 1	KK28	Non Thon Sub-district Health Promotion Hospital	Primary Care Unit	Yes	**No**
Zone 2	KK10	Muengkao Sub-district Health Promotion Hospital	Primary Care Unit	Yes	Yes
Zone 2	KK12	Thapra Sub-district Health Promotion Hospital	Primary Care Unit	**No**	Yes
Zone 2	KK15	Ban Donbom Sub-district Health Promotion Hospital	Primary Care Unit	Yes	Yes
Zone 2	KK16	Don Chang Sub-district Health Promotion Hospital	Primary Care Unit	Yes	Yes
Zone 2	KK29	Don Hun Sub-district Health Promotion Hospital	Primary Care Unit	**No**	**No**
Zone 2	KK30	Banwa Sub-district Health Promotion Hospital	Primary Care Unit	Yes	**No**

Location	Code	Public Healthcare Facility	Service Hierarchy	KKH CUP network	Questionnaire respondent
Zone 2	KK31	Ban Nong Ya Preak Sub-district Health Promotion Hospital	Primary Care Unit	**No**	**No**
Zone 2	KK32	Ban Nong Bua Di Mi Sub-district Health Promotion Hospital	Primary Care Unit	**No**	**No**
Zone 3	KK01	Chatapadung Medical Center (Khon Kaen Hospital)	Primary Care Unit	Yes	Yes
Zone 3	KK06	Hua Thung Primary Care Center	Primary Care Unit	Yes	Yes
Zone 3	KK07	Health Promotion Center 7, Khon Kaen	Specialized Care	**No**	Yes
Zone 3	KK08	Nong Wang Medical Center (Khon Kaen Hospital)	Primary Care Unit	Yes	Yes
Zone 3	KK17	Ban Ped Sub-district Health Promotion Hospital	Primary Care Unit	Yes	Yes
Zone 3	KK21	Khon Kaen Hospital	Tertiary	Yes	Yes
Zone 3	KK22	Khon Kaen Rajanagarindra Psychiatric Hospital	Specialized Care	**No**	Yes
Zone 3	KK25	Pracha Samosorn Medical Center (Khon Kaen Hospital)	Primary Care Unit	Yes	Yes
Zone 4	KK02	Thanyarak Khon Kaen Hospital	Specialized Care	**No**	Yes
Zone 4	KK03	Mittrapap Medical Center (Khon Kaen Hospital)	Primary Care Unit	Yes	Yes
Zone 4	KK04	Ban Thum Sub-district Health Promotion Hospital	Primary Care Unit	Yes	Yes
Zone 4	KK09	Ban Nong Kung Community Health Center	Primary Care Unit	Yes	Yes
Zone 4	KK18	Dang-yai Sub-district Health Promotion Hospital	Primary Care Unit	Yes	Yes
Zone 4	KK19	Sumran Sub-district Health Promotion Hospital	Primary Care Unit	Yes	Yes
Zone 4	KK20	Sawathee Sub-district Health Promotion Hospital	Primary Care Unit	Yes	Yes
Zone 4	KK33	Non Moung Sub-district Health Promotion Hospital	Primary Care Unit	Yes	No
Zone 4	KK34	Ban Kho Sub-district Health Promotion Hospital	Primary Care Unit	Yes	**No**
Zone 4	KK35	Ban Sum Chan Sub-district Health Promotion Hospital	Primary Care Unit	Yes	**No**
Zone 4	KK36	Non Rang Sub-district Health Promotion Hospital	Primary Care Unit	Yes	**No**

Table S2. Details and justifications of Hazard pillar indicators.

Indicators	Sub-indicators	Data source	Rating score
H1: Fluvial Flood	H1.0: Fluvial Flood	Scenario storyline	4 - <=152 - 155 MSL<br>2 - >=152 - 155 MSL<br>0 - >155 MSL
H2: Pluvial Flood	H2.0: Pluvial Flood	Scenario storyline	4 - Possible<br>0 - No possible
H3: Water scarcity	H3.0: Water scarcity	Scenario storyline	4 - Possible<br>0 - No possible

Justifications

- **Fluvial flood (H1.1)**: Corridor of fluvial flood under climate-related hazard bandwidth of Khon Kaen city is used to classify the hazard's magnitude. The occurrence of fluvial flood level <=152 to 155 m MSL is classified as having a high level of the hazard, followed by >=152 to 155 m MSL, and >155 m MSL as medium and low. The flood magnitudes' rating score is proportionately classified as 4, 2, and 0.
- **Pluvial flood (H1.2)**: In this study, the occurrence of a pluvial flood is binary, yes and no. In this regard, the sub-indicator is assorted into 2 classes, (1) possible to experience a pluvial flood, and (2) no possibility to experience a pluvial flood, with rating scores 4 and 0.
- **Water scarcity (H2.0):** The possibility of water scarcity episode is binary, yes or no. Therefore, this sub-indicator is categorized into 2 classes, (1) possible to experience water scarcity and (2) no possibility to experience water scarcity, with rating scores 4 and 0.

Table S3. Details and justifications of Exposure pillar indicators.

Indicators	Sub-indicators	Data source(s)	Rating score
E1: Exposure of public health facility's building(s)	E1.1: Exposure of public health facility's building(s) to fluvial flood	Scenario storyline (GIS analysis)	4 - Flooded or possible to be flooded<br>2 - Not flooded but possible to be isolated or limited accessibility<br>0 - Neither flooded nor be isolated and limited accessibility
	E1.2: Exposure of public health facility's building(s) to pluvial flood	Scenario storyline (GIS analysis)	4 - Located in a repeatedly (pluvial) flooded area<br>2 - Not located a repeatedly (pluvial) flooded area but possible to be flooded due to development-induced landscape/land cover change<br>0 - Neither located in a repeatedly (pluvial) flooded area nor possible to be flooded due to development-induced landscape/land cover change
	E1.3: Exposure of public health facility's building(s) to water scarcity	Scenario storyline (GIS analysis)	4 - Located at 165 MSL and higher,<br>2 - Located lower than 165 MSL<br>0 - Not located in the study area
E2: Exposure of Working systems	E2.1 Exposure of primary working systems	Survey	4 - Located at <3m from the ground level or lower<br>0 - Located at >= 3m from the ground level (2nd floor) or higher
	E2.2 Exposure of secondary working systems	Survey	4 - Located at <3m above the ground or lower<br>0 - Located at >= 3m above the ground or the height of 2nd floor or higher

Justifications

- **Location of public health care building (E 1.0):** Input data of the location of public health facility/building sub-indicator (E1.0) was derived from geospatial analysis of the scenario storylines, which illustrated the presence of public health facilities/buildings to the climate-related hazards as the following:

- *Fluvial flood(E1.1):* Based on the possible bandwidth of fluvial flood scenarios reveals 3 different exposure typologies of the local public health care buildings 1) Flooded or possible to be flooded, 2) Not flooded but possible to be isolated or limited accessibility; 3) Neither flooded nor be isolated and limited accessibility. The rating score assigned to these variations of exposure is 4, 2, and 0, respectively.

- *Pluvial flood(E1.2):* Even though this study cannot identify the spatial extension of the potential pluvial flood area in the future, the current pluvial flood hotspots were taken into account. Therefore, exposure of local public health care buildings to the potential pluvial flood in the future are categorized into 3 levels 1) Located in a repeatedly (pluvial) flooded area, 2) Not located a repeatedly (pluvial) flooded area but possible to be flooded due to development-induced landscape/land cover change, 3) Neither located in a repeatedly (pluvial) flooded area nor possible to be flooded due to development-induced landscape/land cover change. The rating score assigned to these variations of exposure is 4, 2, and 0, respectively. It is important to note that based on conservative assumption, therefore, 0 was not assigned to the participating hospitals.

- *Water scarcity(E1.3)*: Similar to the pluvial flood, this study cannot identify the spatial extension of the potential water scarcity in the future. But the current water intermittent hotspots were taken into account in order to diversify the level of exposure within the study area. Even though there are no current studies or research to specify water scarcity hotspots of the study area, the local water management experts have no objection to using the contour line at 165 m MSL to represent a general topographical limitation of water supply delivery in the study area. Therefore, the local public health facilities' water scarcity exposure classifies into 2 levels, 1) Located at 165 MSL and higher, and 2) Located lower than 165 m MSL, with rating scores 4 and 2, respectively. According to the bandwidth of climate-related hazards, the city is assumed to experience water scarcity as a whole due to network-based water supply. Thereby, no participating hospitals in the study area were assigned as 0 for this sub-indicator.

- **Location/position of primary working systems (E 2.1) and Location/position of and reserved (secondary) working systems (E 2.2):** Exposure of internal 24 primary working systems and 14 secondary working systems (see the list as the remarks below) are determined by its location/position within/outside a local public health facility depending on specific characteristics of the systems in response to flooding. According to the MoPH expert, at least 3 meters from the ground or the 2nd floor of a building is considered as a general recommendation for avoiding flood reaching critical operation systems or equipment. However, a trade-off between disaster risk preparation and appropriateness for day-to-day operation shall be further debated. In this regard, this study assumes that locations of working systems at 3 meters from the ground or 2nd floor or higher of a public health care building is considered as a safer location for essential working systems (rating score 0). In the case that the presence of working systems below 3 meters from the ground level is considered as high exposure with a rating score 4. For the working systems reported as off-site operation and not under the direct control of the health care facility, this study assumes that it is located in a safe location somewhere else (rating score 0). Nevertheless, some critical working system elements could not locate or store in the public health

facility's building due to fire protection or hygiene regulations, e.g. diesel fuels for a backup generator or solid waste/biohazard waste. However, a safer location of these resources needs to be secured and contained to avoid cascading effects. Nonetheless, the study does not include such an extensive elaboration of exposure of critical infrastructures that the local public health depends on into account in this set of composite indicators. However, their dependency and interdependency are clearly addressed in the Vulnerability pillar. It is important to note this study does not include exposure of the working systems to direct or indirect water supply scarcity-related effects due to insufficient supportive input data in the study area.

Remarks:
- List of internal 24 primary working systems: 1) Electricity power control center; 2) Liquid fuel (vehicle, water pumping, cooking etc.); 3) Computer/Server control center; 4) Internet control center; 5) Telephone/Radio control center; 6) Document/Medical record archive; 7) Drinking/ Potable water storage; 8) Water filter or purification system; 9) Tap water; 10) Drainage system; 11) Pumping system;12) Wastewater treatment system;13) Solid waste center; 14) Infectious waste center; 15) Hazardous waste center; 16) Infectious waste incineration point; 17) Medical radiology and imaginary center; 18) Morgue; 19) Food and nutrition storage; 20) Medicine and pharmaceutical storage center; 21) Blood bank; 22)Medical gases and liquid oxygen supply storage/center; 23) Dispensable medical supplies storage; 24) Parking lots
- List of internal 14 secondary working systems: 1) Backup power source (s) (CHP, renewable energy); 2) Liquid fuel (vehicle, water pumping, cooking etc.); 3) Computer/Server control center (including internet); 4) Telephone/Radio control center; 5) Tap water; 6) Water filter/Purification; 7) Wastewater treatment system; 8) Waste management system; 9) Medicine and dispensable medical supplies (incl. Medical gases, blood); 10) Food and nutrition storage; 11) Document/Medical record archive; 12) Multipurpose space/spare room; 13) Morgue; 14) Parking lots

Table S4. Details and justifications of Vulnerability pillar indicators.

Indicators	Sub-indicators	Data source	Vulnerability Rating score
V1: Over carrying capacity	V1.0: Balance between service capacity and service demand	Survey	4 - No, having service demand more than service capacity<br>2 - Yes, having service demand equal to service capacity<br>0 - Yes, having service demand lower than service capacity
V2: Variety of vulnerable patients	V2.0: Variety of vulnerable patient types<br>1) Respiratory ventilator/Oxygen-dependent or Dialysis dependent<br>2) Disability and self-movement difficulty<br>3) Elderly<br>4) Infant/Toddlers (0-5 year-old)<br>5) Pregnancy women<br>6) Long-term medication/treatment dependency<br>7) Local language proficiency<br>8) Mental illness<br>9) Others	Survey	4.0 - >= 8 types<br>3.5 - 7 types<br>3.0 - 6 types<br>2.5 - 5 types<br>2.0 - 4 types<br>1.5 - 3 types<br>1.0 - 2 types<br>0.5 - 1 type<br>0.0 - No vulnerable patients
V3: Resource insufficiency	V3.1: Insufficiency of financial resources for BaU operation	Survey	4 - Insufficient financial resource for BaU operation (deficit)<br>2 - Sufficient financial resource for BaU operation (but no surplus)<br>0 - A surplus financial resource for BaU operation
	V3.2: Insufficiency of health care personnel for BaU operation	Ratio of healthcare personnel per population in a service area or other figures indicated by the interviewees	4 - Yes<br>0 - No
V4: Poor system conditions and	V4.1: System conditions	Survey	4 - Impaired/Dysfunction (Out of order)<br>2 - Fair (O.K.)<br>0 - Good

Indicators	Sub-indicators	Data source	Vulnerability Rating score
maintenance of working systems	V4.2: System monitoring and reporting	Survey	4 – No<br>2 – Yes, but implemented irregularly<br>0 – Yes and implemented regularly
	V4.3: Availability of trained staff/technician for system maintenance and reparation	Survey	4 – No<br>0 – Yes
	V4.4: Availability of maintenance resources	Survey	4 – No<br>2 – Yes, but insufficient<br>0 – Yes and sufficient
V5: Downtime of essential working systems	V5.0: Maximum downtime of essential working systems	Survey	4.00 - >4 days<br>3.43 - >2-4 days<br>2.86 - >1-2 days<br>2.29 - >12-24 hrs<br>1.71 - >4-12 hrs<br>1.14 - >1-4 hrs<br>0.57 - <1 hrs<br>0.00 - Never
V6:Flexibility & modularity	V6.1: Flexibility and modularity of key working systems	Survey	4.00 – Very Low/None<br>2.67 – Low<br>1.33 – Medium<br>0.00 – High<br>**Note:** see the rating scale definition in the justification section
	V6.2: Connectivity(-ability) of essential working system with external devices/systems	Survey	4.00 –None and never aware of it<br>2.67– Low (No, but having a plan/under consideration)<br>1.33 –Medium (Yes, only for particular working systems)<br>0.00 – Very High (Yes, all working systems can be connected with external devices/ systems)
	V6.3: One-stop service area with the highest protective level (in the case of hazards or high level of emergency)	Survey	4 – None and never aware of it<br>3 – Low (No, but under discussion or drafting plan)<br>2 – Medium (Yes, having a plan, but no implementation)<br>1– High (Yes, having a plan, and conducting plan review/ drill(s), but no/insufficient resources for implementation)<br>0 – Very high (Yes, having a plan, conducting plan review/ drill(s), having sufficient resources for implementation)
V7: Diversity of suppliers	V7.0: Diversity of suppliers of key working systems	Survey	4.00 – Very low/none<br>2.67 – Low<br>1.33 – Medium<br>0.00 – High<br>**Note:** see the rating scale definition below
V8: Redundancy	V8.1: Availability and capacity of primary backup systems	Survey	4 – Very low/none<br>3 – Low<br>2 – Medium<br>1 – High<br>0 – Very High<br>**Note:** see the rating scale definition in the justification section
	V8.2: Procurement of special vehicle type (e.g. Boat, amphibian, helicopter, drone) for carrying goods and passengers during emergencies or hazards	Survey	4.00 – None and never aware of it<br>2.67 – Low (no, but having a plan/under consideration)<br>1.33 – Medium (yes, own purchased or contracted service providers but lack of regular technical checkup)<br>0.00 – Very High (yes, own purchased or contracted service providers with regular technical checkup)
	V8.3: Alternate safe accessible route(s)	Survey	4.00 – None and never aware of it

Indicators	Sub-indicators	Data source	Vulnerability Rating score
			2.67 – Low (No, but having a plan/under consideration)<br>1.33 – Medium (yes, surveyed and designed alternate safe accessible route(s) but lack of regular maintenance)<br>0.00 – Very High (yes, surveyed and designed alternate safe accessible route(s) as well as conducting regular maintenance)
	V8.4: Procurement of a secondary backup system	Survey	4.00 – None and never aware of it<br>2.67 – Low (no, but having a plan/under consideration)<br>1.33 – Medium (yes, own purchased or contracted service providers but lack of regular technical checkup)<br>0.00 – Very High (yes, own purchased or contracted service providers with regular technical checkup)
	V8.5: Standard procedure for recording a patient medical data in the case of no IT service	Survey	4 – No<br>0 – Yes, manual (written) recording
	V8.6: Using of runners (courier) as a backup for getting help from outside during communication systems failures	Survey	4 – No<br>0 – Yes
	V8.7: Shelter(s) on-site for staff and family in the case of hazards	Survey	4 – No<br>2 – Yes, but no designated places and supportive facilities in advance<br>0 – Yes with designated places and supportive facilities in advance
	V8.8: Assignment of alternate care site(s)	Survey	4 – None and never aware of it<br>3 – Low (no but under discussion or drafting plan)<br>2 – Medium (Yes, having a plan but no implementation)<br>1– High (Yes, having a plan, conducting plan review/ drill(s), but no/insufficient resources for implementation)<br>0 – Very high (Yes, having a plan, conducting plan review/ drill(s), having sufficient resources for implementation)
V9: Responsiveness	V9.1: Resources conservation plan implementation	Survey	4 – None and never aware of it<br>3 – Low (no, but under discussion or drafting plan)<br>2 – Medium (Yes, having a plan but no regular review and drills)<br>1– High (Yes, having a plan, regular review/drill(s), but no/insufficient resources for implementation)<br>0 – Very high(Yes, having a plan, regular review/drill(s), and having sufficient resources for implementation)
	V9.2: Responsive plan for slow-onset climate-related hazards	Survey	4 – None and never aware of it<br>3 – Low (no but under discussion or drafting plan)<br>2 – Medium (yes, have a plan but no regular review and drills)<br>1– High (yes, have a plan, regular review/drill(s), but no/insufficient resources for implementation)<br>0 – Very high(yes, have a plan, regular review/drill(s), and have sufficient resources for implementation)

Indicators	Sub-indicators	Data source	Vulnerability Rating score
	V9.3: Business continuity plan implementation	Survey	4 – None and never aware of it<br>3 – Low (no, but under discussion or drafting plan)<br>2 – Medium (Yes, having a plan but no regular review and drills)<br>1– High (Yes, having a plan, regular review/drill(s), but no/insufficient resources for implementation)<br>0 – Very high(Yes, having a plan, regular review/drill(s), and having sufficient resources for implementation)
	V9.4: Contingency plan implementation	Survey	4 – None and never aware of it<br>3 – Low (no, but under discussion or drafting plan)<br>2 – Medium (Yes, having a plan but no regular review and drills)<br>1– High (Yes, having a plan, regular review/drill(s), but no/insufficient resources for implementation)<br>0 – Very high(Yes, having a plan, regular review/drill(s), and having sufficient resources for implementation)
	V9.5: Surge personnel capacity plan implementation	Survey	4 – None and never aware of it<br>3 – Low (no, but under discussion or drafting plan)<br>2 – Medium (Yes, having a plan but no regular review and drills)<br>1– High (Yes, having a plan, regular review/drill(s), but no/insufficient resources for implementation)<br>0 – Very high(Yes, having a plan, regular review/drill(s), and having sufficient resources for implementation)
	V9.6: Evacuation plan implementation (both partial and full evacuation)	Survey	4 – None and never aware of it<br>3 – Low (no, but under discussion or drafting plan)<br>2 – Medium (Yes, having a plan but no regular review and drills)<br>1– High (Yes, having a plan, regular review/drill(s), but no/insufficient resources for implementation)<br>0 – Very high(Yes, having a plan, regular review/drill(s), and have sufficient resources for implementation)
	V9.7: Self-help	Survey	4.00 – No plan, no necessary workforce and resources for self-help, only rely on external supports<br>2.67 – Low (Yes, having necessary workforce and resources for initial self-help while waiting for external supports)<br>1.33 – Medium (Yes, having necessary workforce and resources for protecting properties and working system with a minor need for external supports)<br>0.00 – High (Yes, having necessary workforce and resources for protecting properties and working system with no external supports needed)
V10:Resource mobilization	V10.1: Availability and accessibility of financial resources for disaster risk preparation	Survey	4 – No/Insufficient and difficult to acquire the resources from external sources or donation<br>2 –Insufficient but not difficult to acquire the resources from external sources or donation<br>0 – Sufficient and no need to acquire the resources from external sources or donation

Indicators	Sub-indicators	Data source	Vulnerability Rating score
	V10.2: Volunteer and external help management plan implementation	Survey	4 – None and never aware of it<br>3 – Low (no, but under discussion or drafting plan)<br>2 – Medium (Yes, having a plan but no regular review and drills)<br>1– High (Yes, having a plan, regular review/drill(s), but no/insufficient resources for implementation)<br>0 – Very high(Yes, having a plan, regular review/drill(s), and having sufficient resources for implementation)
	V10.3: Availability of resources for reconstruction /reparations and lag time for resuming to full operation	Survey	4.00 – No financial resources<br>2.67 – Low (Yes, having financial resources but >1 year of delay/waiting time for reparation and resuming full operation)<br>1.33 – Medium (Yes, having financial resources but <=6 months of delay/waiting time for reparation and resuming full operation)<br>0.00 – High (Yes, having financial resources but <=1 month of delay/waiting time for reparation and resuming full operation)
V11: Integration and coordination	V11.1: Existence and efficiency of internal Board of committee/working group on disaster risk management	Survey	4.00 – None and never aware of it<br>2.67 – Low (no, but under discussion)<br>1.33 – Medium (Yes, having regular meetings but lack of resources and efficient coordination)<br>0.00 – High (Yes, having regular meetings with sufficient resources and efficient coordination)
	V11.2: Specific coordinator on disaster risk management	Survey	4.00 – None and never aware of it<br>2.67 – Low (no, but under discussion)<br>1.33 – Medium (Yes, having clear designed coordinator (s) but disaster risk management is not his/her main task)<br>0.00 – High (Yes, having clear designed coordinator (s) who disaster risk management is his/her main task)
	V11.3: Patient referral and transfer agreement with other hospitals	Survey	4 – No<br>0 – Yes
	V11.4: Agreement and exercise on partial or full patient evacuation to other hospitals/facilities	Survey	4 – No<br>0 – Yes
	V11.5: Availability of automatically channels or systems for communicating and coordinating with utilities and key suppliers	Survey	4 – No<br>2 – Yes, partially/not all key utilities/suppliers<br>0 – Yes, all key utilities/suppliers
V12: Information	V12.1: Availability and accessibility of local hazard map and climate-related disaster risk database	Survey	4.00 – No and never aware of it<br>2.67 – Low (aware of the information but limited accessibility)<br>1.33 – Medium (aware of and access to the information but does not use it for risk communication with relevant stakeholders)<br>0.00– High (aware of and access to the information and use it for risk communication with relevant stakeholders)
	V12.2: Availability and accessibility of local future population and development projection dataset for long-term service planning	Survey	4.00 – No and never aware of it<br>2.67 – Low (aware of the information but limited accessibility)<br>1.33 – Medium (aware of and access to the information but does not use it for service planning)

Indicators	Sub-indicators	Data source	Vulnerability Rating score
			0.00– High (aware of and access to the information and use it for service planning)
	V12.3: Availability and accessibility of information on local future climate-related disaster risks (floods and water supply scarcity)	Survey	4.00 – No and never aware of it<br>2.67 – Low (aware of the information but limited accessibility)<br>1.33 – Medium (aware of and can be able to access the information but does not use it for risk management planning)<br>0.00– High (aware of and can be able to access the information and use it for risk management planning)
V13: Preparedness and risk transfer	V13.1: Availability plan and long-term investment for increasing climate-related hazards resilience	Survey	4 – None and never aware of it<br>3 – Low (no, but under discussion or drafting plan)<br>2 – Medium (Yes, having a plan but no implementation)<br>1– High (Yes, having a plan, but no/insufficient resources and coordination for implementation)<br>0 – Very high (Yes, having a plan, sufficient resources and coordination for implementation)
	V13.2: Climate-related hazards risk insurance	Survey	4 – None<br>2 – Yes, but not cover all types of climate-related hazards<br>0 – Yes, cover all types of climate-related hazards
	V13.3: Build Back Better plan implementation	Survey	4.00 – None<br>2.67 – Low (no, but under discussion or drafting plan)<br>1.33 – Medium (Yes, having a plan and regular plan review, but no/insufficient resources and coordination for implementation)<br>0.00 – Very high(Yes, having a plan, regular plan review, sufficient resources and coordination for implementation)
V14: Participation and inclusiveness	V14.1: Community participation in disaster risk management planning	Survey	4.00 – Internal process and not involve the community in planning and exercise<br>2.67 – Involve the community in the plan exercise process<br>1.33 – Involve the community in the planning process but irregular exercise the plan with communities<br>0.00 – Involve the community in the planning process and conduct exercises together regularly
	V14.2: Disaster risk management planning and exercise with utilities, suppliers and other relevant agencies	Survey	4 – None and never aware of it<br>3 – Low (no, but under discussion or drafting plan)<br>2 – Medium (Yes, have a plan but no implementation)<br>1– High (Yes, have a plan, but no/insufficient resources and coordination for implementation)<br>0 – Very high(Yes, have a plan, sufficient resources and coordination for implementation)
V15: Capacity development	V15.1: In-house capacity building and awareness-raising on the importance of future climate-related disaster risk and resilience	Survey	4 – None and never aware of it<br>3 – Low (no, but under discussion or drafting plan)<br>2 – Medium (Yes, having a plan but no implementation)

Indicators	Sub-indicators	Data source	Vulnerability Rating score
			1– High (Yes, having a plan, but no/insufficient resources and coordination for implementation)<br>0 – Very high(Yes, having a plan, sufficient resources and coordination for implementation)
	V15.2: Training on working with no-electricity or limited resources	Survey	4 – None and never aware of it<br>3 – Low (no, but under discussion)<br>2 – Medium (yes, <1 time a year)<br>1– High (yes, at least 1 time a year, but having insufficient resources and coordination)<br>0 – Very high (yes, at least 1 time a year and have sufficient resources and coordination)
V16: Mainstreaming climate-risk in planning process	V16.1: Mainstreaming disaster risk management in an action plan or budget plan	Survey	4 – None and never aware of it<br>3 – Low (no, but under discussion or drafting plan)<br>2 – Medium (Yes, having a plan but no implementation)<br>1– High (Yes, having a plan, but no/insufficient resources and coordination for implementation)<br>0 – Very high(Yes, have a plan, sufficient resources and coordination for implementation)
	V16.2: Integration of the future climate-disaster risk information in the system maintenance plan and reparation budget	Survey	4 – None and never aware of it<br>3 – Low (no, but under discussion or drafting plan)<br>2 – Medium (Yes, having a plan but no implementation)<br>1– High (Yes, having a plan, but no/insufficient resources and coordination for implementation)<br>0 – Very high(yes, having a plan, sufficient resources and coordination for implementation)
V17: Monitoring and evaluation	V17.0: Monitoring and evaluation	Survey	4.00 – None<br>2.67 – Low (no, but under discussion or drafting plan)<br>1.33 – Medium (Yes, having a plan and regular plan review, but no/insufficient resources and coordination for implementation)<br>0.00 – Very high(Yes, having a plan, regular plan review, sufficient resources and coordination for implementation)

Justifications

- **Over carrying capacity (V1)**: Many public health care units in Thailand face an overcarrying capacity problem, especially tertiary service-level hospitals. The service demands are usually greater than the number of services/beds available. The impacts of a bed capacity problem can be significant such as downgraded service quality, increased length of time waiting for services, patients &staff satisfaction, and eventually population health at risk [63]. Moreover, limited service capacity interrupts workflows of health care services in both Business as Usual situations and sensitivity to harm under emergency or hazards. In this regard, general self-appraisal on balance between service capacity and service demand is used as a sub-indicator (V1.0).

- **Variety of vulnerable patients (V2)**: [64] defines children, pregnant women, elderly people, malnourished people, and people who are ill or immunocompromised as vulnerable groups who take a relatively high share of the disease burden associated with emergencies and when a disaster strikes. However, [65] categorizes vulnerable groups in the context of Thai's health care based on physical capacity (e.g. pregnant women, elderly, children, physical disability, high acute patients, critical chronic disease

Indicators	Sub-indicators	Data source	Vulnerability Rating score

patient, etc.) and decision autonomy (decision autonomy - e.g. pediatric patients, dementia patients, mental disorder, etc., and limited decision autonomy – e.g. prisoners, enlisted soldiers, illegal immigrants, illegal sex worker, drug users, and etc.). Besides physical perspective, social determinants of health also significantly contribute to health vulnerability (e.g., financial, legal, environmental, and behavioural factors, as well as lacking transportation, substance abuse, and education and literacy issues) [66]. Nevertheless, this study focused on health care operation and management efforts in terms of medically complex and social determinants that cause treatments and services complications during an emergency or hazardous circumstances such as respiratory ventilator/oxygen-dependent, dialysis-dependent patients, disability and self-movement difficulty, elderly, infant/toddlers (0-5-year-old), pregnancy women, medication treatment dependency, local language proficiency, mental illness, and others. The variety and quantity of vulnerable patients determine the complexity of treatment, care, and rehabilitation. However, neither standard nor index is available, which allows comparison among health care units regarding the degree of burden due to the typology and quantity of vulnerable groups. Therefore this study used a variation of vulnerable patients (within a service area) to represent sensitivity elements of health care services. In other words, with more varieties of vulnerable patients under care, a public health care unit must deal with more complex treatments & care and time constraints, especially under a hazard strike.

- **Resource insufficiency(V3):** Conventional resources insufficiency in BaU situation determined climate-related hazards sensitivity of a public health care unit are financial resources and human resources.
  - **Financial resources (V3.1):** In the landscape of Thai's public health care, despite positive outcomes of universal health care coverage, which prevents Thai people go bankrupt due to medical expenses, the state-run hospitals have been facing a financial crisis for almost a decade. In 2017, 558 public hospitals reported financial deficits and huge debts, especially secondary and tertiary hospitals [67]. Financial constraints cause a direct impact on health care operation and delivery of services through difficulties in procurement of resources and payment of running costs, including medical and non-medical supplies, staff salary, payment of utility bills, etc. Moreover, a financial deficit in normal operations puts a public hospital/health care unit in a tight spot during a climate-related crisis. Therefore, financial resources insufficiency of a local public health care unit in usual operation is selected to represent financial sensitivity.
  - **Human resources (V3.2):** Shortage and inequitable distribution of health personnel are the core weaknesses of the Thai public health system. Opposite to the increasing demand for health care services under the universal health care scheme, zero growth in the civil service sector and medical education system cause work overload to the entire health workforce [68]. For example, a nurse in the SHPH provides not only medical treatments and health promotion, she/he is also responsible for administrative tasks such as financial accounting or even security guard. Distracting from the main responsibility of the key health task force may affect patients' service quality and satisfaction. Even though the Thai government put more permanent positions to incentivize the health care workforce who works under COVID-19 crisis, more long-term solutions are still missing. Apparently, personnel shortage has highly impacted the disruption of workflow, which could be worsen under climate-related hazards situations when health personnel have to sustain the service and respond to a disastrous strike at the same time.

- **Poor system conditions and maintenance of working systems (V4):** Ensuring a good condition and well maintenance of essential working systems is paramount to assure continuity of health care service workflow, especially in emergencies. Maintenance also affects several non-core activities of hospitals, such as supplies, accessibility, and security. However, in developing countries, a public health care unit often lacks an engineer /manager who specifically oversights and regularly monitors the systems. Amid emergency or hazards, poor condition and maintenance of essential working systems could put strong pressure

Indicators	Sub-indicators	Data source	Vulnerability Rating score

on a hospital/public health care unit's operation under crisis. Therefore, current system conditions (V4.1), system maintenance monitoring & reporting (V4.2), and availability of trained staff/technicians for system maintenance and reparation (V4.3), as well as the availability of maintenance resources(V4.4), were selected as sub-indicators representing sensitivity aspects of essential working systems. 26 essential working systems were considered in this study as follows: 1) Electricity power control center; 2) backup power source (s) (e.g.CHP, renewable energy); 3) Liquid fuel (for a backup generator, vehicle, water pumping, cooking etc.); 4) Computer/Server control center; 5) Internet control center; 6) Telephone/Radio control center; 7) Document/Medical record archive; 8) Drinking/ Potable water storage; 9) Water filter or purification system; 10) Tap water; 11) Drainage system; 12) Pumping system; 13) Wastewater treatment system; 14) Solid waste center; 15) Infectious waste centre; 16) Hazardous waste center; 17 Infectious waste incineration point; 18) Medical radiology and imaginary center; 19) Morgue; 20) Food and nutrition storage; 21) Medicine and pharmaceutical storage center; 22) Blood bank; 23) Medical gases and liquid oxygen supply storage/center; 24) Dispensable medical supplies storage; 25) Multipurpose space/spare room; 26) Parking lots.

- **Downtime of essential working systems (V5)**: Downtime or outage duration refers to a period of time that a system fails to provide or perform its primary function [69]. Whether it is planned or unplanned, downtime can affect an entire system or may only impact a single application [70]. A non-functioning system can completely paralyze public health care services. This disruption can be fatal if their patient's life relies on the running systems. This time-based threshold (absolute time or relative time) is usually used to track availability loss [71] and monitor resuming time to normal operation. In this study, the maximum downtime experience of each essential working system in the past five years (during 2014-2019) was estimated by the managerial level of a health care facility. This study recognized that the historical downtime records could not directly determine the future disruption of the essential working system, but they can be used as a baseline for the potential impact operationalization. Based on the survey result, an average downtime of each essential working system is benchmarked with the level of effects that occurred to essential working systems, representing a degree of sensitivity-related vulnerability of this study. It is important to note that this research also differentiated the downtime benchmark of different health care service hierarchies.

- **Flexibility & modularity (V6):** The concepts of flexibility and modularity are championed as ways to adapt to future uncertainties [72], especially for the complex system, whereby the system is decomposed into several sub-systems (modules) [73]. Modularity often refers as a characteristic of a system that has functionally interdependence within and independence across modules [74] or imply to a degree of system modules that can be separated, reassembled, scalable, reusable as well as consisting of isolated, self-contained elements [72]. Modularity can be a subset of flexibility, but modularity generally involves creating fixed boundaries of modules and their interfaces. Meanwhile, flexibility has a broader meaning, refers to the ability to change or be changed easily according to the situation or ability to readily adapt without breaking [72,75]. From a resilience perspective, flexibility (and/or modularity) is usually mentioned as the ability to adjust, redistribute, reorganise, restructure, reassemble, and evolve in response to wide-range of changing circumstances of spatially distributed and functionally linked [27-29,31-33,35]. In this study, flexibility & modularity attributes to local public health care represented through three sub-indicators; flexibility & modularity of 18 essential working systems (V6.1) (see description of flexibility and modularity level as below; connectivity (ability) of key working system with external devices/systems (V6.2); and assignment of a one-stop service area with the highest protective level (V6.3).

  Remarks: List of 18 essential working systems -- 1) Electricity power control center; 2) Backup power source (s) (e.g. CHP, renewable energy);3)Computer/Server control center; 4)Internet control center; 5)Telephone/Radio control center; 6)Water filter/Purification; 7)Tap water; 8)Pumping system; 9)Wastewater treatment system; 10)Waste management system (incl. solid waste, infectious waste, hazardous waste); 11)Medicine and dispensable medical supplies (incl. Medical gases, blood); 12) Linin

Indicators	Sub-indicators	Data source	Vulnerability Rating score

service; 13)Food and nutrition; 14)Multipurpose space/spare room; 15)Personnel management; 16)Vehicles; 17)Safe access route; 18)Personnel commuting service

Description of the level of flexibility and modularity of public health care service working systems

Level	Descriptions
**No/insignificant**	The working system can NOT be moved, or adjusted, or modulated its elements or functions.
**Low**	The working system can be moved, adjusted, or modulated its elements or functions by using special equipment/devices or by specialist supervision or specialist supervision. Likely, the working system may have lower efficiency or productivity after moving, adjusting, or modulating its elements or functions.
**Medium**	The working system can be moved, or adjusted, or modulated its elements or functions by specialist supervision or specialist supervision and/or using special equipment/devices. Likely, the working system remains the same level of efficiency or productivity after moving, adjusting, or modulating its elements or functions.
**High**	The working system can be moved, or adjusted, or modulated its elements or functions by users and/or using typical available equipment/devices. Likely, the working system remains the same level of efficiency or productivity after moving, adjusting, or modulating its elements or functions.

- **Diversity of suppliers (V7):** In the resilience perspective, diversity is mentioned as the presence of diversity options, the functionality of systems, networks, operation mode, infrastructures, and resources. However, appraisal of a diversity of functionality of systems, networks and infrastructures required advanced knowledge or specialist to organize the assessment. Nevertheless, a variety of options for resources acquisition for essential working systems could reflect through supplier diversity. Supplier diversity is simple enough and allows self-assessment by the local public health units where usually health care personnel are not trained or well trained as a system engineer or suppliers' manager. It is common for a health care unit to monitor their stockpile and define triggers and thresholds for supplies replenishment. However, looking for alternative sources for maintaining operation during a crisis is still overlooked, especially in areas where never had experience coping with hazards or threats before. Supplier diversity is present when an organization's contracts for goods and services feature various businesses [39]. Besides of benefits of increasing competition and widening the supplier pool, diversity of suppliers is valuable when a health care unit has to deal with emergencies and hazards. At the same time, demands for medical and health care needs are also increased. The efficiency and effectiveness of operational processes in the health care supply chain are expected to reduce the impact of greater losses, especially victims [76]. World major catastrophic events such as COVID-19 pandemic, the Great East Japan Earthquake or the Bangkok flood 2011 proved the great regret of depending on single sourcing. The impacts were cascading down through supply chains especially manufactural facilities, source materials or products from suppliers in risk-prone areas or logistic infrastructure. In this regard, a variety of options for meeting the needs of medical and health services in the health care supply chains is very important. In this study, diversity is considered the lack of supplier diversity of key essential working systems and unreadiness of structures or managerial schemes that allow more than one source of supplies to feed to the operating systems. The description of the level of diversity of suppliers of the 18 public health care essential working systems used for self-assessment of the local public health care unit shows below.

  Remarks: List of 18 essential working systems -- 1) Electricity power control center; 2)Backup power source (s) (e.g. CHP, renewable energy); 3)Computer/Server; 4)Internet; 5)Telephone/Radio; 6)Water filter/Purification; 7)Tap water; 8) Underground

Indicators	Sub-indicators	Data source	Vulnerability Rating score

water; 9)Pumping system; 10)Wastewater treatment system; 11)Solid waste management system (incl. infectious waste, hazardous waste); 12)Medicine and dispensable medical supplies (incl. Medical gases, blood); 13) Linin service; 14) Office stationery; 15)Food and nutrition; 16)Multipurpose space/spare room; 17)Personnel management; 18) Vehicles

Description of the level of diversity of suppliers of public health care essential working systems

Level	Descriptions
**No/insignificant**	Single supplier or monopoly
**Low**	2 or multiple suppliers are available only in the case of an emergency/hazards or business as unusual. No contract and supportive technical structure/management system are agreed upon / installed in advance.
**Medium**	2 or multiple suppliers are available only in the case of emergency/hazards or business as unusual. Contract and supportive technical structure/management system are agreed upon / installed in advance.
**High**	2 or multiple suppliers are available in both emergency/hazards and business as usual. Contract and supportive technical structure/management system are agreed upon and installed in place.

- **Redundancy (V8):** The simplest term for redundancy is a backup or additional of extra components, which become necessary in the case of a mishap or hazard. Spare components or extra resources play a majority role in reducing chances of system failure. Redundancy is very important for risk management of critical systems regarding reliability and availability improvement [77]. Therefore, local public health care units must ensure spare capacity or reserved (back-up) resources/systems in order to cope with shocks, disruptions or surges in demands and be able to sustain operation without external supports or interferences [26,29-30,32-33,35]. This study captured redundancy (availability & capacity) of key 15 working systems in terms of time threshold linked to the level of effects that occurred to public health care service working systems after emergency or disruption begins (V8.1), which evaluated by the local public health care managers. For the system features where temporal measurement does not provide a quantitative redundancy threshold, availability and qualitative measures were checked by the public health care managers, namely alternative types of vehicles (e.g. boat, amphibian, helicopter, drone) (V8.2); alternate safe and accessible route(s) (V8.3); procurement of secondary backup systems (V 8.4); alternate procedure for medical recording(V8.5); options in the case of communication failure(V8.6); shelter for staff and family(8.7); as well as an assignment of alternate care site (V8.8). **Remarks:** List of 15 essential working systems -- 1)Backup power source (s) (e.g. CHP, renewable energy);2) Liquid fuel; 3) Computer/Server 4)Internet; 5)Telephone/Radio; 6)Water filter/Purification; 6)Tap water; 7)Drinking water sources; 8)Wastewater treatment system; 9)Waste management system (incl. solid waste, infectious waste, hazardous waste); 10)Medicine and dispensable medical supplies (incl. Medical gases, blood); 11) Linin service; 12) Office stationery; 13)Food and nutrition; 14)Multipurpose space/spare room; 15)Personnel management

- **Responsiveness (V9):** According to the Cambridge dictionary [75], responsiveness refers to the quality of reacting to something or someone, especially a quick or positive reaction. In the context of climate and disaster risk resilience, responsiveness implies the ability of a system to organize, rearrange and give feedback in a timely manner which allows preparation, warning, and responding actions to shocks and disruptions and restore order to the normal state of operation [27,29,31-35]. From the engineering/computer science or clinic perspective, responsiveness can be quantified *change* over a particular pre-specified time frame [78]. However, this study did not consider the responsiveness of performance or feedback individual sub-system

Indicators	Sub-indicators	Data source	Vulnerability Rating score

or functional unit of public health care. Instead, this research reflects responsiveness through the availability and implementation of plans, enabling a (whole) public health care unit to react to climate-related hazards appropriately and timely. Thus, sub-indicators representing vulnerability caused by responsiveness deficits are the implementations of the following plans; resources conservation plan (V9.1); slow-onset hazards response plan (V9.2); business continuity plan (V9.3); contingency plan (V9.4); surge personnel capacity plan (V9.5); evacuation plan (both partial and full evacuation) (V9.6); and self-reliance capacity (V9.7).

- **Resource mobilization (V10):** Resource mobilization is the ability to secure resources/supports from resources/supports providers and to mobilize them appropriately in order to ensure the continuation of service [79] under shock/stress as well as restore order and function of systems after the math [32-34]. In this regard, a public health care unit needs to acquire financial resources (investment) and workforce (staffing) for coping shocks/disruptions and restoring services from the impact. Therefore, the assessment on the level of availability and accessibility of financial resources for climate-related hazard preparation (V10.1), volunteer and external help management plan (Vc10.2), and availability of resources for reconstruction /reparations against lag time for resuming to full operation (V10.3) are used for indicating resource mobilization.

- **Integration and coordination (V11):** Lillrank [80] summarized that coordination is the arrangement of roles and tasks into an organized whole, while integration is a combination of several specialized and differentiated resources and contributions to create an output that is a system consisting of several parts. Each part needs to contribute to the output, but also submit to the demands of the whole. Both attributes are crucial for public health care operations, either in normal circumstances or under threats. A public health care unit needs to ensure consistency and alignment across relevant operation systems, actors, institutions, networks in order to enable them to function or take collective action based on their interdependency and interconnectedness layers, both within an organization and external stakeholders such as community, utilities, and other disaster risk management agencies. In this regard, this study defined key components representing integration and coordination to minimize the potential impact of climate-related hazards in public health care. Thus, the sub-indicators can be laid out as the followings, existence and efficiency of an internal managerial body on disaster risk management (V11.1), disaster risk management coordinator (V11.2), patient referral and transfer agreement (V11.3), partial or full patient evacuation drills with other hospitals (V11.4), and real-time or almost real-time coordination with utilities and key suppliers (V11.5).

- **Information (V12):** Information plays a central role in disaster risk management. With the advancement of technology, lack of information is no longer a major obstacle for disaster risk reduction [81]. However, in developing countries such as Thailand, data availability and accessibility create huge gaps across development levels, yet mentioning poor data management and integration in both vertical and horizontal layers, especially in disaster risk reduction. This study focuses on capturing basic requirements for the public health care unit to ensure the understanding of the current and anticipatory future of climate change and development change and how these affect their operations and assets, enabling them to strategically work with communities and relevant stakeholders. To this aspect, availability, accessibility, and application of the following information were used to indicate an adaptive capacity element of public health care vulnerability, such as availability and accessibility of hazard map and climate-related hazards database (V12.1), local future population and development for long-term service planning (V12.2), information on local future climate-related hazards in the service area (V12.3).

- **Preparedness and risk transfer(V13):** Preparedness refers to a set of precautionary measures or actions to deal with a potential risk or avoid negative outcomes. Preparedness is good linkages with responsiveness such as early warning, contingency

Indicators	Sub-indicators	Data source	Vulnerability Rating score

planning, stockpiling of equipment and supplies, development of arrangements for coordination, evacuation and public information, and training and field exercises [82]. Nevertheless, ensuring preparedness enhancement for future risk is crucial and needs a long-term planning and investment perspective beyond emergency response. Long-term strategic planning and investment are essential for public health service, not only through minimizing negative outcomes in the emergency management phase but also increased resilience through Build Back Better approach and risk transfer in the recovery phase. To this matter, this study reflects a crucial element of the local public health service vulnerabilities through a deficit in preparedness and risk transfer such as availability plan and long-term investment for increasing climate-related hazards resilience (V13.1), provision of climate-related hazards insurance (V13.2), and build back better plan (V13.3).

- **Participation and inclusiveness (V14):** Quick & Feldman [83] conclude the distinction between participation and inclusion. Participation is collective efforts to increase public input-oriented to the content of programs and policies. Inclusion is creating a community involved in coproducing processes, policies, and programs for defining and addressing public issues with deliberation and diversity. These properties are required for successful disaster risk management. Disaster risk reduction is beyond the capacity of one organization; multi-stakeholders involvement and collaboration are required [84]. Especially, disaster risk management of a public health care unit needs cooperation and support from many relevant actors within and outside their service area boundary in co-producing plan & policies, pooling resources, and conducting the emergency response exercises such as communities, local government, utilities, suppliers, upper administration agencies, urban (spatial) planner, etc. Therefore, lacking or insufficient community participation in disaster risk management planning (V14.1) and inclusiveness of utilities, suppliers and other relevant agencies in disaster risk management planning and exercise (V14.2) were defined as key attributions that amplified vulnerability.

- **Capacity development (V15):** Besides a plenty number of definitions given by international aid agencies, UNDP provides a comprehensive definition of capacity development as capable individuals, organizations and societies which play a vital role in the successful reduction and management of disaster risks [85]. This study focuses on the organizational capacity development of a public health care unit derived from a collective action of individuals in the organization [86]. Therefore, implementing in-house capacity building and awareness-raising on the importance of future climate-related hazards and resilience (V15.1) and provision of training on working with limited resources, e.g. no electricity and no clean water (V15.2), shall be examined to reflect the vulnerability of a local public health care unit. These attributes practically reflect the ability of a public health care unit to internalize knowledge, skills, and experience to improve and transform for dealing with a greater level of unexpected disruptions and retain knowledge over time.

- **Mainstreaming climate risk in planning process (V16):** The recent concept of vulnerability is shifted from a determination of risks towards a future-oriented approach that considers a spectrum of possible futures by taking uncertainties into account [87]. Future-oriented risk-informed planning enables the development of strategic planning on disaster risk reduction based on robust decision making. Therefore, lack of mainstreaming climate-related hazards in a health care unit's action plan and budget plan (V16.1), as well as the integration of future climate risk information in system maintenance and reparation plan (V 16.2), are clear indications of the vulnerability of a local public health care unit under the future climate challenges.

- **Monitoring and evaluation (V17):** Monitoring and evaluation mechanism plays a critical role in defining progress and achievement of disaster risk management policy and actions [88]. Evidence and experiences of ex-ante and ex-post scenarios can help avoid maladaptation, improve learning capacity, and invest efficiently [89]. In this regard, the availability and implementation of

Indicators	Sub-indicators	Data source	Vulnerability Rating score
monitoring and evaluation on disaster risk management (17.0) is a crucial indicator representing a public health care unit's vulnerability.			

Table S5. KMO and Bartlett's Test.

Kaiser-Meyer-Olkin Measure of Sampling Adequacy.		0.524
Bartlett's Test of Sphericity	Approx. Chi-Square	268.260
	df	136
	Sig.	0.000

Table S6. Total variance explained.

Component	Initial Eigenvalues			Extraction Sums of Squared Loadings			Rotation Sums of Squared Loadings[a]
	Total	% of Variance	Cumulative %	Total	% of Variance	Cumulative %	Total
1	6.699	39.406	39.406	6.699	39.406	39.406	6.009
2	1.830	10.763	50.169	1.830	10.763	50.169	1.860
3	1.685	9.910	60.079	1.685	9.910	60.079	1.611
4	1.439	8.465	68.544	1.439	8.465	68.544	1.379
5	1.230	7.237	75.781	1.230	7.237	75.781	3.812
6	.912	5.365	81.146				
7	.808	4.753	85.899				
8	.541	3.180	89.079				
9	.488	2.870	91.949				
10	.393	2.310	94.259				
11	.314	1.844	96.103				
12	.225	1.326	97.429				
13	.179	1.056	98.485				
14	.115	.675	99.159				
15	.095	.559	99.719				
16	.025	.147	99.866				
17	.023	.134	100.000				
Extraction Method: Principal Component Analysis.							
a. When components are correlated, sums of squared loadings cannot be added to obtain a total variance.							

Table S7. Communalities value.

Indicators	Initial	Extraction
V1: Over carrying capacity	1.000	0.751
V2: Variety of vulnerable patients	1.000	0.838
V3: Resource insufficiency	1.000	0.507
V4: Poor system conditions and maintenance of working systems	1.000	0.822
V5: Downtime of sensitive working systems	1.000	0.841
V6: Flexibility & modularity	1.000	0.753
V7: Diversity of suppliers	1.000	0.752
V8: Redundancy	1.000	0.701
V9: Responsiveness	1.000	0.883
V10: Resource mobilization	1.000	0.705
V11: Integration and coordination	1.000	0.688
V12: Information	1.000	0.717
V13: Preparedness and risk transfer	1.000	0.862
V14: Participation and Inclusiveness	1.000	0.717
V15: Capacity development	1.000	0.836
V16: Mainstreaming climate-risk in planning process	1.000	0.785
V17: Monitoring and evaluation	1.000	0.725
Extraction Method: Principal Component Analysis.		

Table S8. Pattern Matrix.

Indicators (variables)	Component				
	1	2	3	4	5
V14	.880				
V17	.849				
V13	.796				
V16	.730				
V15	.725				
V12	.711				
V8	.694				
V9	.625				.467
V11	.524				
V1		.829			
V3		.705			
V7			.809		
V2			.743		
V5				.914	
V10					.808
V6					.801
V4					.800

Extraction Method: Principal Component Analysis.
Rotation Method: Oblimin with Kaiser Normalization.
a. Rotation converged in 13 iterations.

Table S9. Structure Matrix.

Indicators (variables)	Component				
	1	2	3	4	5
V13	.861				.480
V17	.849				
V16	.819				.506
V14	.813				
V15	.803	-.440			.448
V9	.797				.701
V8	.791				.526
V12	.664		.404		
V11	.617	-.432			.409
V1		.829			
V3		.686			
V7			.811		
V2			.744		
V5				.914	
V6	.425				.856
V10					.802
V4			.435		.802

Extraction Method: Principal Component Analysis.
Rotation Method: Oblimin with Kaiser Normalization.

Table S10. Component Correlation Matrix.

Component	1	2	3	4	5
1	1.000	-.062	.046	.025	.347
2	-.062	1.000	.038	-.028	-.133
3	.046	.038	1.000	.005	.073
4	.025	-.028	.005	1.000	.006
5	.347	-.133	.073	.006	1.000

Extraction Method: Principal Component Analysis.
Rotation Method: Oblimin with Kaiser Normalization.

File S1: Questionnaire

Survey for potential impact assessment of climate-related hazards on urban public health service, Khon Kaen city

55 % of the world's population living in urban areas today and is projected to reach 68% by 2050 (UNDESA,2018). Cities expose to climate-related disasters such as flooding, water shortages or extreme weather conditions, imposing huge challenges to urban health facilities and services in the future. Besides ensuring service capabilities to meet growing demands driven by urbanization, the local public health care service must adapt to worsen climate change impacts. In this regard, it is crucial to strengthen climate-resilient urban public health care services by integrating spatial planning and service network operations among urban development domains, disaster risk management and the local health sector.

The purpose of this survey is to assess the potential impact of climate-related hazards on the urban public health service of the Ministry of Public Health in Khon Kaen Province. The survey result will be used for potential impact assessment and climate-resilient operationalization tailored for Khon Kaen city public health service and formulate policy recommendations for mainstreaming integrated climate-resilient and adaption to sectoral and local public health care operation. The responder of this survey should be a responsible person (s) for the environment and safety of the hospital who can coordinate information from various related departments such as management, finance department, maintenance & service support department, nursing, emergency service, and other relevant divisions.

The questionnaire consists of 5 parts as follows

Part 1: General information
Part 2: Status quo – Trend of potential service capacity
Part 3: Sensitivity-related elements
Part 4: Coping capacity-related elements
Part 5: Adaptive capacity-related elements

(Reference: United Nations, Department of Economic and Social Affairs, Population Division (2018). World Urbanization Prospects: The 2018 Revision

- Key Terminology –

Hazard: The potential occurrence of a natural or human-induced physical event or trend or physical impact that may cause loss of life, injury, or other health impacts, as well as damage and loss to property, infrastructure, livelihoods, service provision, ecosystems, and environmental resources. In this report, the term hazard usually refers to climate-related physical events or trends or their physical impacts

Disaster: A serious disruption of the functioning of a community or a society involving widespread human, material, economic or environmental losses and impacts, which exceeds the ability of the affected community or society to cope using its own resources

Exposure: The presence of people, livelihoods, species or ecosystems, environmental functions, services, and resources, infrastructure, or economic, social, or cultural assets in places and settings that could be adversely affected.

Vulnerability: the propensity or predisposition to be adversely affected. Vulnerability encompasses a variety of concepts and elements including sensitivity or susceptibility to harm and lack of capacity to cope and adapt.

Sensitivity: the degree to which a system, person, or community is affected, either adversely or beneficially, by climate-related hazards. Sensitivity is related to physical, socio-economic, culture and environment tendency of a system, person, or community.

Coping capacity The ability of people, institutions, organizations, and systems, using available skills, values, beliefs, resources, and opportunities, to address, manage, and overcome adverse conditions in the short to medium term

Adaptive capacity The ability of systems, institutions, humans, and other organisms to adjust to potential damage, to take advantage of opportunities, or to respond to consequences

Resilience: The capacity of social, economic and environmental systems to cope with a hazardous event or trend or disturbance, responding or reorganizing in ways that maintain their essential function, identity and structure, while also maintaining the capacity for adaptation, learning and transformation

Sources;

IPCC, 2014: Annex II: Glossary [Mach, K.J., S. Planton and C. von Stechow (eds.)]. In: Climate Change 2014: Synthesis Report. Contribution of Working Groups I, II and III to the Fifth Assessment Report of the Intergovernmental Panel on Climate Change [Core Writing Team, R.K. Pachauri and L.A. Meyer (eds.)]. IPCC, Geneva, Switzerland, pp. 117-130.

UNISDR, 2009: UNISDR Terminology on Disaster Risk Reduction. United Nations International Strategy for Disaster Reduction, Geneva, Switzerland.

Part 1: General information

1.1 Organization:……………………………………..……………………………………..…

1.2 Address:…………………………..………….…….……………………………….…..…

1.3 Name-Surname of responder:………..…………….………..……………..………………

1.4 Current designation: ………..……………………..………………………...………..……

1.5 Department/Bureau/Section: ……………………….…………………….….…...……….……

1.6 E-mail: ………………….…….………… 1.7 Telephone: ……………………………….…..

1.8 Gender ☐ Male ☐ Female ☐ Others 1.9 Age: ………………………………..….

1.10 Education level

☐ High school graduate ☐ Bachelor degree or equivalent

☐ Bachelor degree or equivalent ☐ Doctoral degree or equivalent

1.11 Relevant experience/responsibility on risk management or natural disaster risk (if any) management

Year	Description

1.12 Survey response date ……………………………………………………………………………

Part 2: Status quo – Trend of potential service capacity

Note: Please fill in the information or mark ✓ in the box corresponding to characteristics/service potential of your organization.

***** Current operation status (Year 2019) *****

2.1 Please specify the number of patients admitted to your organization in the fiscal year 2019.

Number of emergency patients ………………… persons/day

Number of patients in ………………… persons/ day

Number of outpatients ………………… people /day

Number of patients receiving out-of-office services (e.g. Service unit) ………….. people/day

Others (Please specify ……………………………………………) ………………… People/day

2.2 Do you think that your agency will provide services according to the capacity to accommodate patients according to the health service standards or not?

☐ Yes, the number of patients using the service is equal to the capacity of the service.

☐ No number of patients exceeded the capacity for which the service was available. (Please provide details)

☐ No number of patients using the service is less than the capacity to accommodate. (Please provide details)

*** If you answered "No", please specify the number of patients you think is appropriate. According to the potential to accommodate patients according to the health service standards of your organization (Average estimate)

Number of emergency patients ………………… persons/day

Number of patients in ………………… persons/day

Number of outpatients ………………… people/day

Number of patients receiving out-of-the-box services (e.g. Service unit) ……….. people/day

Others (Please specify …………………………………………….) ……… people/day

2.3 Does your organization have the following types of patients under your current (2019) care or area of care?

Vulnerable patients	No	Yes (Please specify the number of patients)	Remarks
Respiratory ventilator/Oxygen-dependent or Dialysis dependent			
Disability and self-movement difficulty			
Elderly			
Infant/Toddlers (0-5 year-old)			
Pregnancy women			
Continuity medication treatment dependency			
Local language proficiency			
Mental illness patient			
Others (please specify …………………………)			

Notes/comments (if any)

2.4 Please specify the minimum number of personnel for providing service efficiently in accordance with the standards in various situations of your organization as follows:

Service status	Minimum number of personnel (person/day) for operation (Average estimated value)			Remarks
	Medical staff	Medical service staff	Hospital service staff	
Open for service in all departments				
Open part of the service (Only for the work that is necessary)				
Open only for the emergency department / basic laboratory.				
Closed				

Remarks:

- Medical staff: Staff who perform a job that provides medical examination and direct medical treatments, such as doctors, dentists, nurses, nursing assistants, nursing staff, midwives, and other nursing services
- Medical service staff: Staff who perform medical services other than direct medical examinations, such as X-ray personnel, physical therapist, medical technicians, pharmacists, nutritionists and staff members in the medical service department, etc
- Hospital service staff: Staff who perform work not related to medical care or medical services, such as finance and accounting staff, procurement officer, driver, cleaning staff, security guards, etc

Notes/comments (if any)

2.5 Please specify locations of the following essential working systems (more than 1 option is possible)

**Working systems**	**No/not relevant**	**Location of internal working systems**					**Off-site**
		**Outdoor**	**Indoor**				
		**<=1st fl.**	**Under ground**	**1st fl.**	**2nd fl.**	**>2nd fl.**	
Electricity power control center	☐	☐	☐	☐	☐	☐	☐
Back up power source (s) (CHP, renewable energy)	☐	☐	☐	☐	☐	☐	☐
Liquid fuel (for a backup generator, vehicle, water pumping, cooking etc.)	☐	☐	☐	☐	☐	☐	☐
Computer/Server control center	☐	☐	☐	☐	☐	☐	☐
Internet control center	☐	☐	☐	☐	☐	☐	☐
Telephone/Radio control center	☐	☐	☐	☐	☐	☐	☐
Document/Medical record archive	☐	☐	☐	☐	☐	☐	☐
Drinking/ Potable water storage	☐	☐	☐	☐	☐	☐	☐
Water filter or purification system	☐	☐	☐	☐	☐	☐	☐
Water supply (Tap water)	☐	☐	☐	☐	☐	☐	☐
Drainage system	☐	☐	☐	☐	☐	☐	☐
Pumping system	☐	☐	☐	☐	☐	☐	☐
Wastewater treatment system	☐	☐	☐	☐	☐	☐	☐
Solid waste storage	☐	☐	☐	☐	☐	☐	☐
Infectious waste storage	☐	☐	☐	☐	☐	☐	☐
Hazardous waste storage	☐	☐	☐	☐	☐	☐	☐
Infectious waste incineration facility	☐	☐	☐	☐	☐	☐	☐
Medical radiology and imaginary system	☐	☐	☐	☐	☐	☐	☐
Morgue	☐	☐	☐	☐	☐	☐	☐
Food and nutrition storage	☐	☐	☐	☐	☐	☐	☐
Medicine and pharmaceutical storage	☐	☐	☐	☐	☐	☐	☐
Blood bank	☐	☐	☐	☐	☐	☐	☐
Medical gases and liquid oxygen supply storage/center	☐	☐	☐	☐	☐	☐	☐
Dispensible medical supplies storage	☐	☐	☐	☐	☐	☐	☐
Multipurpose space/spare room	☐	☐	☐	☐	☐	☐	☐
Parking lots	☐	☐	☐	☐	☐	☐	☐

Notes/comments (if any)

2.6 The longest downtime/disruption/shortage of the following systems in the past 5 years (2014-2019).

Working systems	Downtime								Not relevant
	Never	<1 hr.	>1-4 hrs.	>4-12 hrs.	>12-24 hrs.	> 2-1 days	>2- 4 days	>4 days	
Grid power control center	☐	☐	☐	☐	☐	☐	☐	☐	☐
Backup power source (s) (e.g. diesel generator, CHP, renewable energy)	☐	☐	☐	☐	☐	☐	☐	☐	☐
Gasoline/Liquid fuel (for a backup generator, vehicle, water pumping, cooking etc.)	☐	☐	☐	☐	☐	☐	☐	☐	☐
Computer/Server control center	☐	☐	☐	☐	☐	☐	☐	☐	☐
Internet control center	☐	☐	☐	☐	☐	☐	☐	☐	☐
Telephone/Radio control center	☐	☐	☐	☐	☐	☐	☐	☐	☐
Drinking/ Potable water	☐	☐	☐	☐	☐	☐	☐	☐	☐
Tap water (water supply)	☐	☐	☐	☐	☐	☐	☐	☐	☐
Pumping system	☐	☐	☐	☐	☐	☐	☐	☐	☐
Wastewater treatment system	☐	☐	☐	☐	☐	☐	☐	☐	☐
Drainage system	☐	☐	☐	☐	☐	☐	☐	☐	☐
Waste management system (solid waste, infectious waste, hazardous waste)	☐	☐	☐	☐	☐	☐	☐	☐	☐
Medicine and dispensable medical supplies (incl. Medical gases, blood)	☐	☐	☐	☐	☐	☐	☐	☐	☐
Linin service	☐	☐	☐	☐	☐	☐	☐	☐	☐
Office stationery	☐	☐	☐	☐	☐	☐	☐	☐	☐
Food and nutrients	☐	☐	☐	☐	☐	☐	☐	☐	☐
Personnel (in charge less than 50 %)	☐	☐	☐	☐	☐	☐	☐	☐	☐
Vehicles	☐	☐	☐	☐	☐	☐	☐	☐	☐
Safe access route(s)	☐	☐	☐	☐	☐	☐	☐	☐	☐
Others (please specify………………..)	☐	☐	☐	☐	☐	☐	☐	☐	☐

Notes/comments (if any)

2.7 How does your organization manage the working conditions and maintenance practice of the following systems?

Working system	System conditions and maintenance				Not relevant
	System condition	Examination & reporting	Trained staff/technician	Resources	
Grid power system (electricity)	☐ Disrepair<br>☐ Fair<br>☐ Good	☐ None<br>☐ Yes, but irregular<br>☐ Yes and regular	☐ None<br>☐ Yes	☐ None<br>☐ Yes, but insufficient<br>☐ Yes and sufficient	☐
Backup power source (s) (e.g. diesel generator, CHP, renewable energy)	☐ Disrepair<br>☐ Fair<br>☐ Good	☐ None<br>☐ Yes, but irregular<br>☐ Yes and regular	☐ None<br>☐ Yes	☐ None<br>☐ Yes, but insufficient<br>☐ Yes and sufficient	☐
Gasoline (liquid) storage	☐ Disrepair<br>☐ Fair<br>☐ Good	☐ None<br>☐ Yes, but irregular<br>☐ Yes and regular	☐ None<br>☐ Yes	☐ None<br>☐ Yes, but insufficient<br>☐ Yes and sufficient	☐
Computer/Server control center	☐ Disrepair<br>☐ Fair<br>☐ Good	☐ None<br>☐ Yes, but irregular<br>☐ Yes and regular	☐ None<br>☐ Yes	☐ None<br>☐ Yes, but insufficient<br>☐ Yes and sufficient	☐
Internet control center	☐ Disrepair<br>☐ Fair<br>☐ Good	☐ None<br>☐ Yes, but irregular<br>☐ Yes and regular	☐ None<br>☐ Yes	☐ None<br>☐ Yes, but insufficient<br>☐ Yes and sufficient	☐
Telephone/Radio control center	☐ Disrepair<br>☐ Fair<br>☐ Good	☐ None<br>☐ Yes, but irregular<br>☐ Yes and regular	☐ None<br>☐ Yes	☐ None<br>☐ Yes, but insufficient<br>☐ Yes and sufficient	☐
Water filter or purification system	☐ Disrepair<br>☐ Fair<br>☐ Good	☐ None<br>☐ Yes, but irregular<br>☐ Yes and regular	☐ None<br>☐ Yes	☐ None<br>☐ Yes, but insufficient<br>☐ Yes and sufficient	☐
Water supply (Tap water)	☐ Disrepair<br>☐ Fair	☐ None	☐ None<br>☐ Yes	☐ None	☐

Working system	System conditions and maintenance				Not relevant
	System condition	Examination & reporting	Trained staff/technician	Resources	
	☐ Good	☐ Yes, but irregular<br>☐ Yes and regular		☐ Yes, but insufficient<br>☐ Yes and sufficient	
Pumping system	☐ Disrepair<br>☐ Fair<br>☐ Good	☐ None<br>☐ Yes, but irregular<br>☐ Yes and regular	☐ None<br>☐ Yes	☐ None<br>☐ Yes, but insufficient<br>☐ Yes and sufficient	☐
Wastewater treatment system	☐ Disrepair<br>☐ Fair<br>☐ Good	☐ None<br>☐ Yes, but irregular<br>☐ Yes and regular	☐ None<br>☐ Yes	☐ None<br>☐ Yes, but insufficient<br>☐ Yes and sufficient	☐
Drainage system	☐ Disrepair<br>☐ Fair<br>☐ Good	☐ None<br>☐ Yes, but irregular<br>☐ Yes and regular	☐ None<br>☐ Yes	☐ None<br>☐ Yes, but insufficient<br>☐ Yes and sufficient	☐
Waste management system (solid waste, infectious waste, hazardous waste)	☐ Disrepair<br>☐ Fair<br>☐ Good	☐ None<br>☐ Yes, but irregular<br>☐ Yes and regular	☐ None<br>☐ Yes	☐ None<br>☐ Yes, but insufficient<br>☐ Yes and sufficient	☐
Document/Medical record archive	☐ Disrepair<br>☐ Fair<br>☐ Good	☐ None<br>☐ Yes, but irregular<br>☐ Yes and regular	☐ None<br>☐ Yes	☐ None<br>☐ Yes, but insufficient<br>☐ Yes and sufficient	☐
Linin service	☐ Disrepair<br>☐ Fair<br>☐ Good	☐ None<br>☐ Yes, but irregular<br>☐ Yes and regular	☐ None<br>☐ Yes	☐ None<br>☐ Yes, but insufficient<br>☐ Yes and sufficient	☐
Office stationery	☐ Disrepair<br>☐ Fair<br>☐ Good	☐ None<br>☐ Yes, but irregular<br>☐ Yes and regular	☐ None<br>☐ Yes	☐ None<br>☐ Yes, but insufficient<br>☐ Yes and sufficient	☐

**Working system**	**System conditions and maintenance**				**Not relevant**
	**System condition**	**Examination & reporting**	**Trained staff/technician**	**Resources**	
Vehicles	☐ Disrepair<br>☐ Fair<br>☐ Good	☐ None<br>☐ Yes, but irregular<br>☐ Yes and regular	☐ None<br>☐ Yes	☐ None<br>☐ Yes, but insufficient<br>☐ Yes and sufficient	☐
Safe access route(s)	☐ Disrepair<br>☐ Fair<br>☐ Good	☐ None<br>☐ Yes, but irregular<br>☐ Yes and regular	☐ None<br>☐ Yes	☐ None<br>☐ Yes, but insufficient<br>☐ Yes and sufficient	☐
Others (please specify………………..)	☐ Disrepair<br>☐ Fair<br>☐ Good	☐ None<br>☐ Yes, but irregular<br>☐ Yes and regular	☐ None<br>☐ Yes	☐ None<br>☐ Yes, but insufficient<br>☐ Yes and sufficient	☐

Notes/comments (if any)

2.8 Has your agency ever experienced an emergency or disaster in the past 5 years (2014-2019)? If yes, how has it affected to work systems and operations of your organization?

☐ Never

☐ Yes, but all systems can still work/perform continuously without significant impact.

☐ Yes, the hospital had to temporarily reduce/suspense some services for…… day(s).

☐ Yes, the hospital can only open critical service sections and announce partial evacuation of patients/staff or relocation of essential working systems to a safer location for ………. day(s).

☐ Yes, the hospital had shut down all work systems, announced full evacuation and relocation of essential working systems to a safer location for……. day(s)

If you answered "Yes", please provide a brief description of the incident (s)

………

2.9 Are there differences of patients with a particular type of illness or diseases during hazard events (especially floods or water supply shortages (drought)) and business as usual situations?

☐ No

☐ Yes, please specify details in the table below.

Flooding	Water supply shortages (drought)

Notes/comments (if any)

<u>**Forecasting future conditions**</u>

2.10 If in the next 18 years (2037) (by the end of the 20-year National Strategy's timeframe), there is an increasing number of health population/patients in your service areas by 60% compared to today situation (2019). Are there needs of your organization to expand or increase a service capacity or units in response to the increased number of patients or not?.and how?

☐ None, please specify the reason briefly

☐ Yes, in the same area / same location

- Please specify a number of beds / service units and / or buildings ………………
- Please specify characteristics /functions of the potential units/buildings

☐ Yes, in other locations

- Please specify a number of beds / service units and / or buildings ………………
- Please specify characteristics /functions of the potential units/buildings
- Please provide details the potential units/buildings locations (e.g. address, village, sub-district)

Part 3: Sensitivity-related elements

Please appraise the level of impact of working systems in your organization if confronted with the following situations without external help or supports in accordance with the given potential downtime.

Table 3-1 Description of the level of impacts that occurred to public health care service working systems

Impact level	Descriptions
**Very low**	All services can continue without any discernible impact or change
**Low**	Some services may be reduced or suspended.<br>Some advanced or special services may be canceled.<br>Services for non-priority client/section may be temporarily suspended.
**Medium**	Shutdown of auxiliaries sections, but most critical service may continue.<br>Obtain needed resources/supports from outside<br>Full implementation of conservation measures in order to sustain essential services<br>Limit new inflow patients and maximize patient discharge.<br>Declare total diversion status or partial or total evacuation.
**High**	Discontinued of service to most critical services<br>No new patients' admission.<br>All patients will be transferred to others/nearby service facility<br>Declare total evacuation.<br>Only designed staff assigned to control and observe loss and damages allow stay on-site

3.1 Power system is outage or failure (both from the main power system and the backup system)

Potential downtime	Impact level (see table 3-1)				Not relevant (no system in place)
	Very low	Low	Medium	High	
< 1 hr.	☐	☐	☐	☐	☐
>1-4 hrs.	☐	☐	☐	☐	☐
>4-12 hrs.	☐	☐	☐	☐	☐
>12-24 hrs.	☐	☐	☐	☐	☐
>1-2 days	☐	☐	☐	☐	☐
> 4-2 days	☐	☐	☐	☐	☐
> 4 days	☐	☐	☐	☐	☐

3.2 Shortage of water supply (both tap water and reserved storage)

Potential downtime	Impact level (see table 3-1)				Not relevant (no system in place)
	Very low	Low	Medium	High	
< 1 hr.	☐	☐	☐	☐	☐
>1-4 hrs.	☐	☐	☐	☐	☐
>4-12 hrs.	☐	☐	☐	☐	☐
>12-24 hrs.	☐	☐	☐	☐	☐
>1-2 days	☐	☐	☐	☐	☐
> 4-2 days	☐	☐	☐	☐	☐
> 4 days	☐	☐	☐	☐	☐

3.3 **Shortage of Gasoline/Liquid fuel** (for a backup generator, vehicle, water pumping, cooking, etc.)

Potential downtime	Impact level (see table 3-1)				Not relevant (no system in place)
	Very low	Low	Medium	High	
< 1 hr.	☐	☐	☐	☐	☐
>1-4 hrs.	☐	☐	☐	☐	☐
>4-12 hrs.	☐	☐	☐	☐	☐
>12-24 hrs.	☐	☐	☐	☐	☐
>1-2 days	☐	☐	☐	☐	☐
> 4-2 days	☐	☐	☐	☐	☐
> 4 days	☐	☐	☐	☐	☐

3.4 Computer and server system disruption or failure

**Potential downtime**	**Impact level (see table 3-1)**				**Not relevant (no system in place)**
	**Very low**	**Low**	**Medium**	**High**	
< 1 hr.	☐	☐	☐	☐	☐
>1-4 hrs.	☐	☐	☐	☐	☐
>4-12 hrs.	☐	☐	☐	☐	☐
>12-24 hrs.	☐	☐	☐	☐	☐
>1-2 days	☐	☐	☐	☐	☐
> 4-2 days	☐	☐	☐	☐	☐
> 4 days	☐	☐	☐	☐	☐

3.5 Internet system disruption or failure

**Potential downtime**	**Impact level (see table 3-1)**				**Not relevant (no system in place)**
	**Very low**	**Low**	**Medium**	**High**	
< 1 hr.	☐	☐	☐	☐	☐
>1-4 hrs.	☐	☐	☐	☐	☐
>4-12 hrs.	☐	☐	☐	☐	☐
>12-24 hrs.	☐	☐	☐	☐	☐
>1-2 days	☐	☐	☐	☐	☐
> 4-2 days	☐	☐	☐	☐	☐
> 4 days	☐	☐	☐	☐	☐

3.6 Telephone/radio system disruption or failure

**Potential downtime**	**Impact level (see table 3-1)**				**Not relevant (no system in place)**
	**Very low**	**Low**	**Medium**	**High**	
< 1 hr.	☐	☐	☐	☐	☐
>1-4 hrs.	☐	☐	☐	☐	☐
>4-12 hrs.	☐	☐	☐	☐	☐
>12-24 hrs.	☐	☐	☐	☐	☐
>1-2 days	☐	☐	☐	☐	☐
> 4-2 days	☐	☐	☐	☐	☐
> 4 days	☐	☐	☐	☐	☐

3.7 Waste management system (including solid waste, infectious waste, hazardous waste) disruption or failure

Potential downtime	Impact level (see table 3-1)				Not relevant (no system in place)
	Very low	Low	Medium	High	
< 1 hr.	☐	☐	☐	☐	☐
>1-4 hrs.	☐	☐	☐	☐	☐
>4-12 hrs.	☐	☐	☐	☐	☐
>12-24 hrs.	☐	☐	☐	☐	☐
>1-2 days	☐	☐	☐	☐	☐
> 4-2 days	☐	☐	☐	☐	☐
> 4 days	☐	☐	☐	☐	☐

3.8 Wastewater treatment system disruption or failure f

Potential downtime	Impact level (see table 3-1)				Not relevant (no system in place)
	Very low	Low	Medium	High	
< 1 hr.	☐	☐	☐	☐	☐
>1-4 hrs.	☐	☐	☐	☐	☐
>4-12 hrs.	☐	☐	☐	☐	☐
>12-24 hrs.	☐	☐	☐	☐	☐
>1-2 days	☐	☐	☐	☐	☐
> 4-2 days	☐	☐	☐	☐	☐
> 4 days	☐	☐	☐	☐	☐

3.9 Shortage of food, nutrients and drinking water

Potential downtime	Impact level (see table 3-1)				Not relevant (no system in place)
	Very low	Low	Medium	High	
< 1 hr.	☐	☐	☐	☐	☐
>1-4 hrs.	☐	☐	☐	☐	☐
>4-12 hrs.	☐	☐	☐	☐	☐
>12-24 hrs.	☐	☐	☐	☐	☐
>1-2 days	☐	☐	☐	☐	☐
> 4-2 days	☐	☐	☐	☐	☐
> 4 days	☐	☐	☐	☐	☐

3.10 Shortage of medicine and dispensable medical supplies (incl. medical gases, blood, etc.)

Potential downtime	Impact level (see table 3-1)				Not relevant (no system in place)
	Very low	Low	Medium	High	
< 1 hr.	☐	☐	☐	☐	☐
>1-4 hrs.	☐	☐	☐	☐	☐
>4-12 hrs.	☐	☐	☐	☐	☐
>12-24 hrs.	☐	☐	☐	☐	☐
>1-2 days	☐	☐	☐	☐	☐
> 4-2 days	☐	☐	☐	☐	☐
> 4 days	☐	☐	☐	☐	☐

3.11 Shortage of linin or disruption of linin service

Potential downtime	Impact level (see table 3-1)				Not relevant (no system in place)
	Very low	Low	Medium	High	
< 1 hr.	☐	☐	☐	☐	☐
>1-4 hrs.	☐	☐	☐	☐	☐
>4-12 hrs.	☐	☐	☐	☐	☐
>12-24 hrs.	☐	☐	☐	☐	☐
>1-2 days	☐	☐	☐	☐	☐
> 4-2 days	☐	☐	☐	☐	☐
> 4 days	☐	☐	☐	☐	☐

3.12 Shortage of office stationery

Potential downtime	Impact level (see table 3-1)				Not relevant (no system in place)
	Very low	Low	Medium	High	
< 1 hr.	☐	☐	☐	☐	☐
>1-4 hrs.	☐	☐	☐	☐	☐
>4-12 hrs.	☐	☐	☐	☐	☐
>12-24 hrs.	☐	☐	☐	☐	☐
>1-2 days	☐	☐	☐	☐	☐
> 4-2 days	☐	☐	☐	☐	☐
> 4 days	☐	☐	☐	☐	☐

3.13 Shortage of 50% of staff (compared to BaU)

**Potential downtime**	**Impact level (see table 3-1)**				**Not relevant (no system in place)**
	**Very low**	**Low**	**Medium**	**High**	
< 1 hr.	☐	☐	☐	☐	☐
>1-4 hrs.	☐	☐	☐	☐	☐
>4-12 hrs.	☐	☐	☐	☐	☐
>12-24 hrs.	☐	☐	☐	☐	☐
>1-2 days	☐	☐	☐	☐	☐
> 4-2 days	☐	☐	☐	☐	☐
> 4 days	☐	☐	☐	☐	☐

3.14 No vehicles and safe accessible route (s)

**Potential downtime**	**Impact level (see table 3-1)**				**Not relevant (no system in place)**
	**Very low**	**Low**	**Medium**	**High**	
< 1 hr.	☐	☐	☐	☐	☐
>1-4 hrs.	☐	☐	☐	☐	☐
>4-12 hrs.	☐	☐	☐	☐	☐
>12-24 hrs.	☐	☐	☐	☐	☐
>1-2 days	☐	☐	☐	☐	☐
> 4-2 days	☐	☐	☐	☐	☐
> 4 days	☐	☐	☐	☐	☐

Notes/comments (if any)

Part 4: Coping capacity element

4.1 (current) Level of flexibility and modularity of essential working systems

Table 4-1 Description of flexibility and modularity levels of essential working systems

Level	Descriptions
**No/insignificant**	The working system can NOT be moved, or adjusted, or modulated its elements or functions.
**Low**	The working system can be moved, or adjusted, or modulated its elements or functions by using special equipment/devices or done by specialist supervision or specialist supervision. Likely, the working system may have lower efficiency or productivity after moving, adjusting, or modulating its elements or functions.
**Medium**	The working system can be moved, or adjusted, or modulated its elements or functions by specialist supervision or specialist supervision and/or using special equipment/devices. Likely, the working system remains the same level of efficiency or productivity after moving, adjusting, or modulating its elements or functions.
**High**	The working system can be moved, or adjusted, or modulated its elements or functions by users and/or using typical available equipment/devices. Likely, the working system remains the same level of efficiency or productivity after moving, adjusting, or modulating its elements or functions.

**Working systems**	**Level of flexibility and modularity (see Table 4-1)**				**Not relevant (no system in place)**
	**No**	**Low**	**Medium**	**High**	
Grid (electricity) power control center	☐	☐	☐	☐	☐
Backup power source (s) (e.g. diesel generator, CHP, renewable energy)	☐	☐	☐	☐	☐
Computer/Server control center	☐	☐	☐	☐	☐
Internet control center	☐	☐	☐	☐	☐
Telephone/Radio control center	☐	☐	☐	☐	☐
Water filter/Purification	☐	☐	☐	☐	☐
Water supply (Tap water)	☐	☐	☐	☐	☐
Pumping system	☐	☐	☐	☐	☐
Wastewater treatment system	☐	☐	☐	☐	☐
Waste management system (incl. solid waste, infectious waste, hazardous waste)	☐	☐	☐	☐	☐
Medicine and dispensable medical supplies (incl. medical gases, blood, etc.)	☐	☐	☐	☐	☐
Linin service	☐	☐	☐	☐	☐
Office stationery	☐	☐	☐	☐	☐
Food and nutrition	☐	☐	☐	☐	☐

Working systems	Level of flexibility and modularity (see Table 4-1)				Not relevant (no system in place)
	No	Low	Medium	High	
Multipurpose space/spare room	☐	☐	☐	☐	☐
Personnel management	☐	☐	☐	☐	☐
Vehicles	☐	☐	☐	☐	☐
Safe access route	☐	☐	☐	☐	☐
Personnel commuting service	☐	☐	☐	☐	☐
Others (please specify……………….)	☐	☐	☐	☐	☐

Notes/comments (if any)

4.2 (current) Level of diversity of suppliers which necessary for essential working systems

Table 4-2 Description of diversity of suppliers' levels

Level	Descriptions
**No/insignificant**	Single supplier or monopoly
**Low**	Two or multiple suppliers are available only in the case of an emergency/hazards or business as unusual. No contract and supportive technical structure/management system are agreed upon and installed in advance.
**Medium**	Two or multiple suppliers are available only in the case of emergency/hazards or business as unusual. Contract and supportive technical structure/management system are agreed upon and installed in advance.
**High**	Two or multiple suppliers are available in both emergency/hazards and business as usual. Contract and supportive technical structure/management system are agreed upon and installed in place.

Working systems	Level of diversity of suppliers (see Table 4-2)				Not relevant (no system in place)
	No	Low	No	Low	
Grid (electricity) power control center	☐	☐	☐	☐	☐
Backup power source (s) (diesel generator, CHP, renewable energy, etc.)	☐	☐	☐	☐	☐
Computer/Server control center	☐	☐	☐	☐	☐
Internet control center	☐	☐	☐	☐	☐
Telephone/Radio control center	☐	☐	☐	☐	☐
Water filter/Purification	☐	☐	☐	☐	☐
Water supply (Tap water)	☐	☐	☐	☐	☐
Underground water	☐	☐	☐	☐	☐

**Working systems**	**Level of diversity of suppliers (see Table 4-2)**				**Not relevant (no system in place)**
	**No**	**Low**	**No**	**Low**	
Pumping system	☐	☐	☐	☐	☐
Wastewater treatment system	☐	☐	☐	☐	☐
Waste management system (solid waste, infectious waste, hazardous waste)	☐	☐	☐	☐	☐
Medicine and dispensable medical supplies (incl. Medical gases, blood)	☐	☐	☐	☐	☐
Linin service	☐	☐	☐	☐	☐
Office stationery	☐	☐	☐	☐	☐
Food and nutrition	☐	☐	☐	☐	☐
Multipurpose space/spare room	☐	☐	☐	☐	☐
Personnel management	☐	☐	☐	☐	☐
Vehicles	☐	☐	☐	☐	☐
Personnel commuting service	☐	☐	☐	☐	☐
Others (please specify………………)	☐	☐	☐	☐	☐

Notes/comments (if any)

4.3 Potential redundancy of essential working systems in the case that primary systems/resources malfunction and no replenishment/replacement is possible

**Working system**	**No**	**Reserved systems or resources which can be used after primary systems malfunction**					
		**1-0hr**	**>1-4 hrs**	**>4-12 hrs**	**> 2-1 days**	**> 4-2 days**	**>4 days**
Backup power source (s) (diesel generator, CHP, renewable energy, etc.)	☐	☐	☐	☐	☐	☐	☐
Fuel (for diesel generators, vehicles, pumping, etc.)	☐	☐	☐	☐	☐	☐	☐
Computer/server (including reserved batteries)	☐	☐	☐	☐	☐	☐	☐
Internet	☐	☐	☐	☐	☐	☐	☐
Telephone/Radio (including reserved batteries)	☐	☐	☐	☐	☐	☐	☐
Water supply	☐	☐	☐	☐	☐	☐	☐
Drinking water	☐	☐	☐	☐	☐	☐	☐
Water filter/Purification	☐	☐	☐	☐	☐	☐	☐
Water storage system (e.g. pond, tank)	☐	☐	☐	☐	☐	☐	☐
Pumping system	☐	☐	☐	☐	☐	☐	☐
Wastewater treatment system	☐	☐	☐	☐	☐	☐	☐

**Working system**	**No**	**Reserved systems or resources which can be used after primary systems malfunction**					
		**1-0hr**	**>1-4 hrs**	**>4-12 hrs**	**> 2-1 days**	**> 4-2 days**	**>4 days**
Waste management system (solid waste, infectious waste, hazardous waste)	☐	☐	☐	☐	☐	☐	☐
Medicine and dispensable medical supplies (incl. Medical gases, blood)	☐	☐	☐	☐	☐	☐	☐
Linin service	☐	☐	☐	☐	☐	☐	☐
Office stationery	☐	☐	☐	☐	☐	☐	☐
Food and nutrients	☐	☐	☐	☐	☐	☐	☐
Multipurpose space/spare room	☐	☐	☐	☐	☐	☐	☐
Surge/backup personnel	☐	☐	☐	☐	☐	☐	☐
Others (please specify………………)	☐	☐	☐	☐	☐	☐	☐

Notes/comments (if any)

4.4 Positions/locations of backup working systems/resources

**Working systems**	**No/not relevant**	**Location of internal working systems**					**Off-site**
		**Outdoor**	**Indoor**				
		**<=1st fl.**	**Under ground**	**1st fl.**	**2nd fl.**	**> 2nd fl.**	
Backup power source (s) (diesel generator, CHP, renewable energy, etc.)	☐	☐	☐	☐	☐	☐	☐
Liquid fuel (for a backup generator, vehicle, water pumping, cooking etc.)	☐	☐	☐	☐	☐	☐	☐
Computer/server (including reserved batteries)	☐	☐	☐	☐	☐	☐	☐
Telephone/Radio (including reserved batteries)	☐	☐	☐	☐	☐	☐	☐
Water supply	☐	☐	☐	☐	☐	☐	☐
Water filter/Purification	☐	☐	☐	☐	☐	☐	☐
Wastewater treatment system	☐	☐	☐	☐	☐	☐	☐
Waste management system (solid waste, infectious waste, hazardous waste)	☐	☐	☐	☐	☐	☐	☐
Water storage system (e.g. pond, tank)	☐	☐	☐	☐	☐	☐	☐
Pumping system	☐	☐	☐	☐	☐	☐	☐
Medicine and dispensable medical supplies (incl. Medical gases, blood)	☐	☐	☐	☐	☐	☐	☐
Food and nutrients	☐	☐	☐	☐	☐	☐	☐

Working systems	No/not relevant	Location of internal working systems					Off-site
		Outdoor	Indoor				
		<=1st fl.	Under ground	1st fl.	2nd fl.	> 2nd fl.	
Document/Medical record archive	☐	☐	☐	☐	☐	☐	☐
Multipurpose space/spare room	☐	☐	☐	☐	☐	☐	☐
Morgue	☐	☐	☐	☐	☐	☐	☐
Parking lots	☐	☐	☐	☐	☐	☐	☐
Others (please specify………………)	☐	☐	☐	☐	☐	☐	☐

Notes/comments (if any)

4.5 Procurement of special vehicle type (e.g. Boat, amphibian, helicopter, drone) for carrying goods and passengers during emergencies or hazards

☐ None and never aware of it

☐ No, but have a plan/under consideration)

☐ Yes, own purchased or contracted service providers but lack of regular technical checkup)

☐ Yes, own purchased or contracted service providers with regular technical checkup)

4.6 Alternate safe accessible route(s)

☐ None and never aware of it

☐ No, but have a plan/under consideration)

☐ Yes, surveyed and designed alternate safe accessible route(s) but lack of regular maintenance

☐ Yes, surveyed and designed alternate safe accessible route(s) and conducted regular maintenance

4.7 Connectivity(-ability) of sensitive working system with external devices/systems

☐ None and never aware of it

☐ No, but have a plan/under consideration

☐ Yes, only particular working systems

☐ Yes, all working systems can connect with external devices or systems

4.8 Procurement of a secondary backup system

☐ None and never aware of it

☐ No, but have a plan/under consideration)

☐ Yes, own purchased or contracted service providers but lack of regular technical checkup

☐ Yes, own purchased or contracted service providers with a regular technical checkup

4.9 Resources conservation plan implementation

☐ None and never aware of it

☐ No, but under discussion or drafting plan

☐ Yes, have a plan but no regular review and drills

☐ Yes, have a plan, regular review/drill(s), but no/insufficient resources for implementation

☐ Yes, have a plan, regular review/drill(s), and have sufficient resources for implementation

4.10 Shelter(s) for staff and family in the case of hazards

☐ No

☐ Yes, but no designated places and facilities in advance

☐ Yes, with designated places and facilities in advance

4.11 Patient referral and transfer agreement with other hospitals

☐ No

☐ Yes

If yes, please identify the designated referral hospitals

1)
2)
3)

4.12 Agreement and exercise on partial or full patient evacuation to other hospitals/facilities in the case of emergency or hazards

☐ No

☐ Yes

If yes, please identify the designated referral hospitals

1)
2)
3)

4.13 Standard procedure for recording a patient medical data in the case of no computer service

☐ No

☐ Yes, manual (written) recording

4.14 Using of runners (courier) as a backup for getting help from outside during communication systems failures

☐ No

☐ Yes

4.15 Availability of automatically channels or systems for communicating and coordinating with utilities and key suppliers

☐ No

☐ Yes, partially/not all key utilities/suppliers

☐ Yes, all key utilities/suppliers

4.16 Responsive plan for slow-onset natural hazards

☐ None and never aware of it

☐ No, but under discussion or drafting plan

☐ Yes, have a plan but no regular review and drills

☐ Yes, have a plan, regular review/drill(s), but no/insufficient resources for implementation

☐ Yes, have a plan, regular review/drill(s), and have sufficient resources for implementation

4.17 Self-help capacity

☐ No plan, no necessary workforce and resources for self-help, only rely on external supports

☐ Yes, have necessary workforce and resources for initial self-help while waiting for external supports

☐ Yes, have necessary workforce and resources for protecting properties and working system with a little needs for external support)

☐ Yes, have necessary workforce and resources for protecting properties and working system with no external supports needed)

4.18 Availability and accessibility financial resources for BaU operation

☐ Insufficient financial resource for BaU operation (deficit)

☐ Sufficient financial resource for BaU operation (but no surplus)

☐ Surplus financial resource for BaU operation

4.19 Availability and accessibility of financial resources for disaster risk preparation

☐ No/Insufficient and difficult to acquire the resources from external sources or donation

☐ Insufficient but not difficult to acquire the resources from external sources or donation

☐ Sufficient and no need to acquire the resources from external sources or donation

4.20 Availability and accessibility of BaU financial resources or other sources (e.g. donation, emergency budget from central, provincial, and local governments) for long-term investment on increasing climate-related hazards resilience

☐ No/Insufficient and difficult to acquire the resources from external sources or donation

☐ Insufficient but not difficult to acquire the resources from external sources or donation

☐ Sufficient and no need to acquire the resources from external sources or donation

Notes/comments (if any)

Part 5 Adaptive capacity

5.1 Availability and accessibility of local future population and development for long-term service planning

☐ No and never aware of it
☐ Aware of the information but limited accessibility
☐ Aware of and access to the information but does not use it for service planning
☐ Aware of and access to the information and use it for service planning

5.2 Availability and accessibility of information on local future climate-related disaster risks (floods and water supply scarcity) in your area

☐ No and never aware of it
☐ Aware of the information but limited accessibility
☐ Aware of and access to the information but does not use it for risk management planning
☐ Aware of and access to the information and use it for risk management planning

5.3 Availability and accessibility of local hazard map and climate-related disaster risk database

☐ No and never aware of it
☐ Aware of the information but limited accessibility
☐ Aware of and access to the information but does not use it for risk communication with relevant stakeholders
☐ Aware of and access to the information and use it for risk communication with relevant stakeholders

5.4 In-house capacity building and awareness-raising on the importance of future climate-related disaster risk and resilience

☐ None and never aware of it
☐ No, but under discussion or drafting plan
☐ Yes, have a plan but no implementation
☐ Yes, have a plan, but no/insufficient resources and coordination for implementation
☐ Yes, have a plan, sufficient resources and coordination for implementation

5.5 Business continuity plan implementation

☐ None and never aware of it
☐ No, but under discussion or drafting plan
☐ Yes, have a plan but no regular review and drills
☐ Yes, have a plan, regular review/drill(s), but no/insufficient resources for implementation
☐ Yes, have a plan, regular review/drill(s), and have sufficient resources for implementation

5.6 Contingency plan implementation

☐ None and never aware of it
☐ No, but under discussion or drafting plan
☐ Yes, have a plan but no regular review and drills
☐ Yes, have a plan, regular review/drill(s), but no/insufficient resources for implementation
☐ Yes, have a plan, regular review/drill(s), and have sufficient resources for implementation

5.7 Existence and efficiency of internal Board of committee/working group on disaster risk management
☐ None and never aware of it
☐ No, but under discussion)
☐ Yes, have regular meetings but lack of resources and efficient coordination
☐ Yes; have regular meetings with sufficient resources and efficient coordination

5.8 Specific coordinator on disaster risk management
☐ None and never aware of it
☐ No, but under discussion
☐ Yes, have a clear designed coordinator (s), but disaster risk management is not his/her main task
☐ Yes, have a clear designed coordinator (s) who disaster risk management is his/her main task

5.9 Surge personnel capacity plan implementation
☐ None and never aware of it
☐ No, but under discussion or drafting plan
☐ Yes, have a plan but no regular review and drills
☐ Yes, have a plan, regular review/drill(s), but no/insufficient resources for implementation
☐ Yes, have a plan, regular review/drill(s), and have sufficient resources for implementation

5.10 Training on working with no-electricity or limited resources
☐ None and never aware of it
☐ No, but under discussion
☐ Yes, <1 time a year
☐ Yes, at least 1 time a year, but have insufficient resources and coordination
☐ Yes, at least 1 time a year and have sufficient resources and coordination

5.11 One-stop service area with the highest protective level, in the case of hazards or high level of emergency
☐ None and never aware of it
☐ No, but under discussion or drafting plan
☐ Yes, have a plan, but no implementation
☐ Yes, have a plan, conduct plan review/ drill(s), but no/insufficient resources for implementation
☐ Yes, have a plan, conduct plan review/ drill(s), have sufficient resources for implementation

5.12 Assignment of alternate care site(s)
☐ None and never aware of it
☐ No, but under discussion or drafting plan
☐ Yes, have a plan but no implementation
☐ Yes, have a plan, conduct plan review/ drill(s), but no/insufficient resources for implementation
☐ Yes, have a plan, conduct plan review/ drill(s), have sufficient resources for implementation)

5.13 Evacuation plan implementation (both partial and full evacuation)
☐ None and never aware of it

- ☐ No, but under discussion or drafting plan
- ☐ Yes, have a plan but no regular review and drills)
- ☐ Yes, have a plan, regular review/drill(s), but no/insufficient resources for implementation)
- ☐ Yes, have a plan, regular review/drill(s), and have sufficient resources for implementation)

5.14 Volunteer and external help management plan implementation

- ☐ None and never aware of it
- ☐ No, but under discussion or drafting plan)
- ☐ Yes, have a plan but no regular review and drills)
- ☐ Yes, have a plan, regular review/drill(s), but no/insufficient resources for implementation)
- ☐ Yes, have a plan, regular review/drill(s), and have sufficient resources for implementation)

5.15 Community participation in disaster risk management planning

- ☐ Internal process and not involve the community in planning and exercise process
- ☐ Involve the community in the plan exercise process
- ☐ Involve the community in the planning process but irregular exercise the plan with communities
- ☐ Involve the community in the planning process and exercise regularly

5.16 Disaster risk management planning and exercise with utilities, suppliers and other relevant agencies

- ☐ None and never aware of it
- ☐ No, but under discussion or drafting plan
- ☐ Yes, have a plan but no implementation
- ☐ Yes, have a plan, but no/insufficient resources and coordination for implementation
- ☐ Yes, have a plan, sufficient resources and coordination for implementation

5.17 Mainstreaming disaster risk management in an action plan or budget plan

- ☐ None and never aware of it
- ☐ No, but under discussion or drafting plan
- ☐ Yes, have a plan but no implementation
- ☐ Yes, have a plan, but no/insufficient resources and coordination for implementation
- ☐ Yes, have a plan, sufficient resources and coordination for implementation

5.18 Availability of climate-related hazards management integration on action plans and BaU budget plans

- ☐ None and never aware of it
- ☐ No, but under discussion or drafting plan
- ☐ Yes, have a plan but no implementation
- ☐ Yes, have a plan, but no/insufficient resources and coordination for implementation
- ☐ Yes, have a plan, sufficient resources and coordination for implementation

5.19 Build Back Better plan implementation

- ☐ None
- ☐ No, but under discussion or drafting plan

☐ Yes, have a plan and regular plan review, but no/insufficient resources and coordination for implementation)
☐ Yes, have a plan, regular plan review, sufficient resources and coordination for implementation)

5.20 Monitoring and evaluation
☐ None
☐ No, but under discussion or drafting plan
☐ Yes, have a plan and regular plan review, but no/insufficient resources and coordination for implementation
☐ Yes, have a plan, regular plan review, sufficient resources and coordination for implementation

5.21 Availability of BaU resources for reconstruction /reparations and lag time for resuming to full operation
☐ No financial resources
☐ Yes, have financial resources but >1 year of delay for reparation and resuming full operation
☐ Yes, have financial resources but <=6 months of delay for reparation and resuming full operation
☐ Yes, have financial resources but <=1 month of delay for reparation and resuming full operation

5.22 Integration of the future climate-disaster risk impact information in the system maintenance plan and reparation budget
☐ None and never aware of it
☐ No, but under discussion or drafting plan
☐ Yes, have a plan but no implementation
☐ Yes, have a plan, but no/insufficient resources and coordination for implementation
☐ Yes, have a plan, sufficient resources and coordination for implementation

5.23: Climate-related hazards risk insurance
☐ None
☐ Yes, but not cover all types of climate-related hazards
☐ Yes, cover all types of climate-related hazards

5.24 Shall MoPH's hospital be insured on climate-related hazards? Please justify your support argumentation

Please return the questionnaire in *.doc, *.pdf, or *.jpg format to below contact information

Email wiriya.puntub@tu-dortmund.de By 29 February 2019

Thank you very much for your kind cooperation

Wiriya Puntub
